# Repurposing antimicrobials with ultrasound-triggered nanoscale systems for targeted biofilm drug delivery

**DOI:** 10.1038/s44259-025-00086-3

**Published:** 2025-04-01

**Authors:** Victor Choi, Dario Carugo, Eleanor Stride

**Affiliations:** 1 Department of Engineering Science, Institute of Biomedical Engineering, University of Oxford, Oxford, UK; 2 Nuffield Department of Orthopaedics, Rheumatology and Musculoskeletal Sciences, University of Oxford, Oxford, UK

**Keywords:** Drug delivery, Antimicrobial resistance

## Abstract

Chronic infections represent a major clinical challenge due to the enhanced antimicrobial tolerance of biofilm-dwelling bacteria. To address this challenge, an ultrasound-responsive nanoscale drug delivery platform (nanodroplets) is presented in this work, loaded with four different antimicrobial agents, capable of simultaneous biofilm disruption and targeted antimicrobial delivery. When loaded, a robust protective effect against clinically-derived MRSA and ESBL Gram-positive and Gram-negative planktonic isolates was shown in vitro. Upon application of therapeutic ultrasound, an average 7.6-fold, 44.4-fold, and 25.5-fold reduction was observed in the antibiotic concentrations compared to free drug required to reach the MBC, MBEC and complete persister eradication levels, respectively. Nanodroplets substantially altered subcellular distribution of encapsulated antimicrobials, enhancing accumulation of antimicrobials by 11.1-fold within the biofilm-residing bacteria’s cytoplasm compared to treatment with unencapsulated drugs. These findings illustrate the potential of this multifunctional platform to overcome the critical penetration and localization limitations of antimicrobials within biofilms, opening potential new avenues in the treatment of chronic clinical infections.

## Introduction

Chronic and nosocomial clinical infections, notoriously resistant to traditional antimicrobial therapies, are often perpetuated by bacterial biofilms – complex, dynamic communities that thrive in a self-constructed exopolysaccharide (EPS) matrix^1,2^. This matrix, which is largely impermeable to conventional antibiotics, can foster non-metabolic dormant cells and enable bacterial communication (quorum sensing). Consequently, many traditional antimicrobials are ineffective against biofilms at conventional dosages, necessitating invasive surgical interventions (e.g., in diabetic ulcers) or lifelong high-dose antibiotic regimes (e.g., in cystic fibrosis and urinary tract infections). The holy grail of antibiofilm therapies has thus far eluded researchers, with most agents failing to effectively penetrate and accumulate beyond the biofilm matrix, succumbing to rapid clearance and nuclease-mediated degradation. While antibiofilm drug delivery systems have gained traction due to the long and costly process of developing new drugs^3^, many fail to eradicate biofilm structures completely, allowing new microbial colonization and perpetuating chronic infections^4^.

Our study aims to address this challenge by pioneering the use of an antimicrobial-loaded ultrasound-responsive nanoscale delivery platform (Fig. S1). By harnessing the power of therapeutic focused ultrasound (FUS), we demonstrate simultaneous spatiotemporally controlled antimicrobial release and biofilm disruption. By incorporating existing antimicrobials into this platform, we overcome the limitations of conventional gas-filled microbubbles, which are thwarted by their large size and poor perfusion of the microvasculature^5^. Particularly in antibiofilm therapy, they are rapidly destroyed by the ultrasound pressures needed for mechanical biofilm structure disruption^6^ and activation of metabolically inactive “dormant” cells^7^. Volatile liquid “nano” droplets, designed to vaporize upon ultrasound exposure, transiently form these microbubbles that synergistically disrupt biofilm structures and enhance convective transport through acoustic cavitation (i.e., volumetric bubble oscillations), while ensuring precise, localized drug delivery.

Building on promising, yet incomplete, co-delivery strategies whose efficacy is constrained by drug transport through the biofilm^8,9,10^, we instead directly incorporate four distinct classes of antimicrobial agents separately within a nanodroplet formed from a modified clinical contrast agent (Definity RT™). This strategy leverages the physicochemical stabilization and penetration afforded by the nanoscale carrier and enables simultaneous localized drug delivery and biofilm disruption, thus providing a promising solution for combating chronic and recalcitrant bacterial infections.

## Results and discussion

### Antimicrobial-loaded nanodroplet platform development and characterization

Phospholipid-coated nanodroplets were synthesized via an established procedure^11^ based on a clinically approved contrast agent (Definity RT™) and separately loaded with four antimicrobials with distinct mechanisms of antibiofilm action: a ruthenium polypyridyl complex (antimicrobial metal^12^; Ru-NDs) embedded in the lipid shell, azithromycin (quorum sensing inhibitor^13^; AZM-NDs) and besifloxacin (fluoroquinolone^14^; BES-NDs) in the hydrophobic region, and polymyxin B (antimicrobial peptide^15^; PMB-NDs) electrostatically bound to the anionic shell.

While antimicrobial metals have attracted attention for their therapeutic potential, none have been clinically approved for systemic antibacterial treatment. For this study, a modified ruthenium complex was developed^16–19^, and coupled with a host phospholipid through a strain promoted alkyne-azide cycloaddition (SPAAC) “click” reaction. The successful synthesis and purification of the complex were confirmed through NMR and HPLC-ESI-MS (m/z [M]+: 792.27). Parent complex identity was further verified by evaluating the metal-to-ligand charge-transfer (MLCT) absorption and corresponding fluorescence emission at 456 nm and 624 nm, respectively (Fig. S2)^20^.

**Fig. 1:**
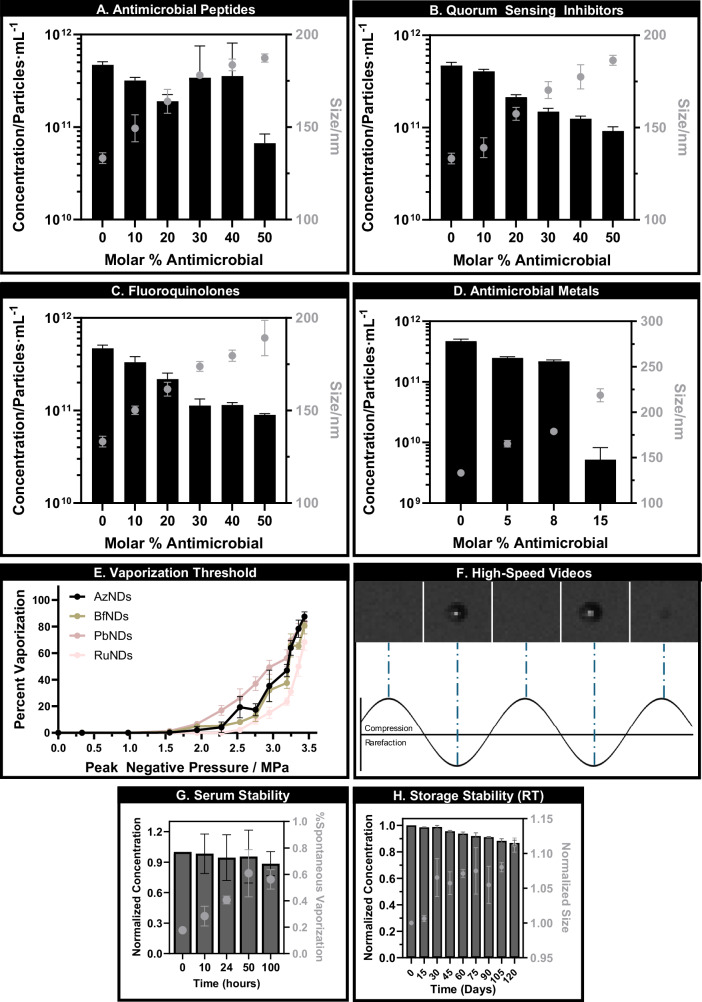
Physicochemical characterization of antimicrobial-loaded nanodroplets. Nanodroplet size and concentration characterization of polymyxin B nanodroplets (**A**; PMB-NDs), azithromycin nanodroplets (**B**; AZM-NDs), besifloxacin nanodroplets (**C**; BES-NDs), and ruthenium polypyridyl nanodroplets (**D**; Ru-NDs) assessed through interferometry, dynamic light scattering, and electro-impedance volumetric zone sensing. Concentration (black bars) and size (gray symbols) values were obtained by matching particle counts at 402 nm across measurement techniques. Formulated nanodroplets were evaluated for their vaporization threshold (**E**) using ultra-high-speed recording (10 MFPS) following exposure to 3.125 MHz ultrasound at varying acoustic pressures (between 0 and 3.4 MPa peak negative pressure). Example images of droplet vaporization captured across the first cycles are represented in (**F**), overlayed on an example sinusoidal ultrasound pulse. Both size and concentration of nanodroplets were conserved for up to 100 h at 37 °C in serum (**G**) and 120 days at room temperature (**H**), showing less than 20% reduction in concentration. All results are presented as the average ± standard deviation across five independent samples, with two technical replicates each.

Microbubbles and nanodroplets were characterized using interferometry, dynamic light scattering (DLS), and electro-impedance volumetric zone sensing (Coulter Counter; aperture size range: 0.4–12 µm) (Fig. 1A–D) to portray the full particle population. Size and concentration of the nanodroplets were carefully controlled, with mean hydrodynamic diameters ranging from 125 nm to 250 nm, significantly smaller than their microbubble counterparts (350–650 nm) due to condensation of the perfluorocarbon gaseous core into the liquid state^21^ (Fig. S3). When loading antimicrobials onto the nanodroplet platform, a clear concentration-dependent relationship could be observed with nanodroplet characteristics, with the appearance of heterogeneity suggesting the disruption of structural integrity at higher concentrations^22^. Concentrations beyond 40 mol% displayed a significant decrease in nanodroplet concentration and an increase in size and polydispersity index (PDI, i.e., a measure of particle size dispersity), indicative of nanodroplet instability and unsuitability for biofilm disruption^23^. A 40 mol% loading concentration was thus selected to balance the maximization of antimicrobial content while maintaining the structural integrity, ultrasound responsiveness, and stability of nanodroplets. Due to lipid concentration limits with the phospholipid-conjugated ruthenium complex, only a maximum 8 mol% could successfully be loaded for the ruthenium-loaded nanodroplets. Critically, all 40 mol% antimicrobial-loaded nanodroplets exhibited less than a 20% reduction in concentration over 100 h in serum and 120 days at room temperature (Fig. 1G, H; Figs. S4, S5)^24^.

Using a custom-designed ultra-high speed imaging setup, nanodroplet vaporization dynamics were observed in response to 3.125 MHz ultrasound (3 pulses of 10 cycles with a 3.2 µs envelope at 37 °C) from a clinical curved array probe, revealing their expansion and contraction behavior in real-time. At peak negative pressures (pnp) above 3.1 ± 0.42 MPa, the initially unresolvable nanodroplets expanded near the trough of each rarefactional half-cycle before contracting during the compressional half-cycle (Fig. 1F, Supplementary Video 1)^25^, resulting in the formation of microbubbles with a mean diameter of 0.9 ± 0.14 µm. This phenomenon is consistent with the hypothesized 5-6x radial expansion factor of perfluorocarbon droplets^26^.

### Clinical isolate planktonic activity of antimicrobial-loaded nanodroplets

A panel of ten clinical bacterial isolates was evaluated, comprising *Escherichia coli* strains (including extended spectrum beta-lactamase (ESBL)-producing and non-ESBL phenotypes) and *Staphylococcus aureus* strains (both MRSA and MSSA variants) sourced from clinical investigations of urinary tract infections, osteomyelitis, and endocarditis in Oxfordshire, UK (Fig. S7)^27,28^. Planktonic antimicrobial susceptibility testing was performed using three measures: minimum inhibitory concentrations (MICs) following EUCAST protocols, metabolic MICs using resazurin in physiologically-relevant media (synthetic human urine^29^ for *E. coli* and brain heart infusion for *S. aureus*), and minimum bactericidal concentrations (MBCs) of stationary-phase populations via standard plating methodologies.

**Fig. 2:**
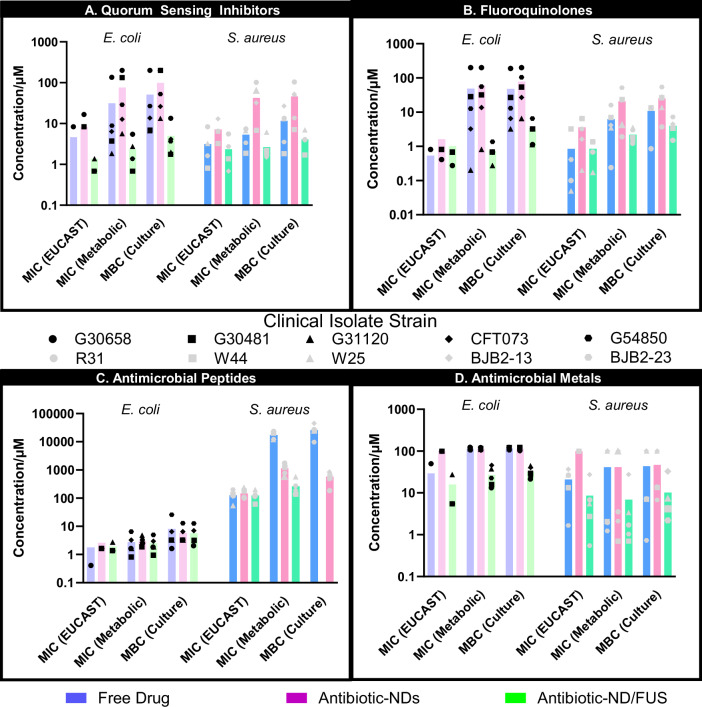
Planktonic antimicrobial efficacy of antimicrobial-loaded ND/FUS. Planktonic antimicrobial activity of free drug (blue bars), antimicrobial-loaded nanodroplets (purple/pink bars), and antimicrobial-loaded ND/FUS (green bars) against *E. coli* (lighter colors) and *S. aureus* (darker colors) clinical isolates across azithromycin (AZM-NDs; **A**), besifloxacin (BES-NDs; **B**), polymyxin B (PMB-NDs; **C**), and ruthenium polypyridyl complex (Ru-NDs; **D**). Plots depict the concentration (µM) of antimicrobial required to achieve the Minimum Inhibitory Concentration (MIC) per EUCAST standards and metabolic viability (resazurin), Minimum Bactericidal Concentration (MBC; defined as a 3-log reduction in CFU/mL). Each colored symbol corresponds to a different clinical isolate strain, as identified in the middle legend, with each isolate represented by three biological replicates, each with three technical replicates per measurement technique.

Azithromycin (Fig. 2A), a broad-spectrum macrolide, exhibited anticipated inhibitory and bactericidal efficacy in its unencapsulated form against both *E. coli* and *S. aureus*^30–35^. AZ-NDs demonstrated reduced bioavailability, necessitating elevated concentrations (3.23-fold for metabolic MIC (*p* = 0.008; Log(BF_10_) = 2.1) and 2.31-fold for MBC (*p* = 0.029; Log(BF_10_) = 1.1)) to achieve equivalent endpoints—an expected consequence of the protective nanodroplet formulation. The addition of focused ultrasound (AZ-ND/FUS; 3.125 MHz, alternating vaporization (3.2 MPa pnp, effective “on” of 3.2 µs/pulse) and delivery (332.9 kPa pnp, effective “on” of 25 µs/pulse) ultrasound) markedly potentiated azithromycin efficacy, reducing metabolic MIC and MBCs for *E. coli* by a factor of 13.45 and 19.18, respectively, compared to free azithromycin, and by 32.71 and 37.19, compared to AZ-NDs alone. Analogous trends were observed in *S. aureus*, where MBCs were reduced with AZ-ND/FUS by 2.84-fold (*p* = 0.073; Log(BF_10_) = 0.7) and 11.14-fold (*p* = 0.035; Log(BF_10_) = 1.2) relative to free azithromycin and AZ-NDs alone, respectively. Interestingly, MIC determinations under EUCAST guidelines showed no significant improvement in *S. aureus* despite observations in metabolic MIC and MBC, emphasizing the need for multiple measures of efficacy.

Besifloxacin (Fig. 2B), a fourth-generation ophthalmic fluoroquinolone with broad-spectrum activity, demonstrated superior in vitro potency among the tested antimicrobials, consistent with previous studies^36,37^. Nanodroplet encapsulation produced modest attenuation of activity, with slight increases in MIC and MBCs compared to free drug (1.63-fold for *E. coli* MBC (*p* = 0.063; Log(BF_10_) = 0.8) and 2.32-fold for *S. aureus* MBC (*p* = 0.060; Log(BF_10_) = 0.8)). However, the addition of focused ultrasound (BF-ND/FUS) markedly enhanced besifloxacin’s bactericidal activity, with substantial reductions in metabolic MIC (66.05-fold for *E. coli* (*p* = 0.138; Log(BF_10_) = 0.2) and 2.81-fold for *S. aureus* (*p* = 0.094; Log(BF_10_) = 0.5)) and MBC (15.72-fold for *E. coli* (*p* = 0.141; Log(BF_10_) = 0.2) and 2.71-fold for *S. aureus* (*p* = 0.014; Log(BF_10_) = 1.9)) relative to free besifloxacin. Notably, BF-ND/FUS overcame intrinsic fluoroquinolone resistance in an *E. coli* isolate (G30658), reducing MIC and MBC requirements by 292-fold and 169-fold, respectively, relative to free drug. However, as with azithromycin, BF-ND/FUS showed reduced efficacy relative to free drug in both species through MIC testing under EUCAST guidelines.

Polymyxin B (Fig. 2C), an antimicrobial peptide targeting Gram-negative bacteria, exhibited robust activity against *E. coli* isolates, with MIC and MBC values consistent with established breakpoints^34,35^. Interestingly, encapsulating PMB in nanodroplets (PMB-NDs) did not significantly attenuate bactericidal activity or growth inhibition compared to free drug in *E. coli* clinical isolates (*p* = 0.245; log(BF_10_) = 0.3 for metabolic MIC; *p* = 0.205; log(BF_10_) = 0.2 for MBC), potentially due to the electrostatic mechanism by which it was attached. While both polymyxin B and ultrasound target bacterial membranes^38^, and would be expected to have a synergistic mechanistic effect^39^, it is possible that ultrasound accelerates the formation of transmembrane pores, allowing polymyxin B to enter bacterial cells and travel beyond its target membrane site, thus reducing its effects against planktonic isolates. However, in *S. aureus* clinical isolates, a bacterium intrinsically resistant to polymyxin B, PMB-ND/FUS decreased metabolic MIC and MBC concentrations by 3.3-fold (*p* = 0.011; log(BF_10_) = 2.1) and 2.7-fold (*p* = 0.011; log(BF_10_) = 2.0), respectively, compared to free drug, demonstrating retained synergy in this Gram-positive pathogen. These results suggest that PMB-ND/FUS retains its ability to potentiate activity even in resistant bacterial strains.

The ruthenium polypyridyl complex (Fig. 2D) exhibited comparatively modest antimicrobial activity, requiring the highest concentrations to achieve MIC and MBC thresholds for both *E. coli* and *S. aureus*. Ru-NDs did not confer a significant protective effect when evaluating metabolic MIC and MBC values but did show a slight protective effect under EUCAST-defined MIC testing when compared to free drug. Regardless, the addition of FUS (Ru-ND/FUS) significantly lowered all three measures by an average of 3.46 (*p* < 0.001; log(BF_10_) = 7.6) and 5.96-fold (*p* = 0.007; log(BF_10_) = 2.2) compared to free drug, in *E. coli* and *S. aureus* isolates, respectively, demonstrating its potential to enhance ruthenium’s efficacy.

Taken together, these data demonstrate that nanodroplet encapsulation provides consistent protection, increasing MBC requirements by an average of 3.08-fold compared to free drug (*p* < 0.001; log(BF_10_) = 4.9). FUS application generates robust potentiation, reducing the antibiotic concentrations needed to attain the MIC and MBC by 12.67-fold (*p* < 0.001; log(BF_10_) = 6.4) and 7.64-fold (*p* < 0.001; log(BF_10_) = 7.0), respectively, likely via acoustic cavitation-mediated membrane permeabilization facilitating improved penetration and intracellular uptake^36,37^. These findings establish ND/FUS as an effective strategy for potentiating diverse antimicrobial classes against both Gram-positive and Gram-negative pathogens.

### Clinical isolate antibiofilm activity of antimicrobial-loaded nanodroplets

Biofilm matrices are highly susceptible to re-colonization following eradication^40^, necessitating strategies that target both the extracellular matrix and viable bacterial populations. Conventional plate count techniques, which require complete biofilm removal, fail to adequately account for matrix contributions and the viable but non-culturable (VBNC) state in biofilms, limiting their applicability for biofilm studies^41^. To overcome these limitations, a combination of assays measuring biomass, metabolic viability, and culturability were employed to determine the minimum biofilm eradication concentration (MBEC) of 72 h mature biofilms, quantifying the lowest antimicrobial concentration required to eliminate biomass, metabolically viable cells^42^, or culturable bacteria. Ultrasound (FUS) alone, applied under the tested parameters, exhibited no intrinsic antimicrobial efforts but reduced biomass by an average of 31.6% across isolates, consistent with previous reports of high-intensity FUS disrupting biofilm EPSs^43^. Similarly, unloaded nanodroplets alone did not exhibit intrinsic antibiofilm activity when measured through culture; however, when applied undiluted, they demonstrated a modest capacity (< 30%) for biofilm dispersal which was contingent on media and strain. Although this dispersal effect did not result in measurable cell death within biofilms, undiluted droplets did yield an approximate 20% reduction in cell viability specifically against the BJB2-13 and BJB2-23 osteomyelitis isolates, with no detectable antimicrobial effects observed in other isolates. Lipid suspensions showed no antibiofilm or antimicrobial activity. Both phenomena are likely attributable to the spontaneous vaporization of formed nanodroplets, likely driven by the inherent instability of low-boiling-point perfluorocarbon droplets as a factor of temperature and interfacial tension^44^.

**Fig. 3:**
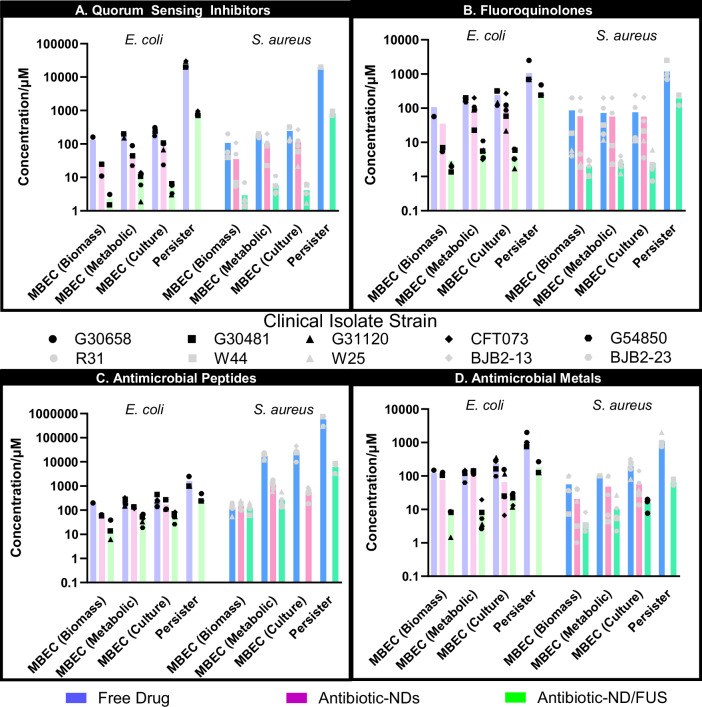
Antibiofilm activity of antimicrobial-loaded ND/FUS. Antibiofilm activity of free drug (blue bars), antimicrobial-loaded nanodroplets (purple/pink bars), and antimicrobial-loaded ND/FUS (green bars) against *E. coli* (lighter colors) and *S. aureus* (darker colors) clinical isolates across azithromycin (AZM-NDs; **A**), besifloxacin (BES-NDs; **B**), polymyxin B (PMB-NDs; **C**), and ruthenium polypyridyl complex (Ru-NDs; **D**). Plots depict the concentration (µM) of antimicrobial required to achieve the Minimum Biofilm Eradication Concentration (MBEC). MBEC metrics include biomass reduction (90% reduction in safranin), metabolic viability (80% reduction in resazurin) and culturability (3-log reduction in CFU/mL). Persister assays are conducted and defined as the lowest concentration needed for complete eradication below the detection limit of 10° CFU/mL. Each colored symbol corresponds to a different clinical isolate strain, with each isolate represented by three biological replicates, each with three technical replicates per measurement technique. Due to the excess antimicrobial concentration required for complete bacterial eradication with antimicrobial-loaded nanodroplets alone, persister elimination tests and MBEC culture tests in PMB-NDs alone against *S. aureus* were not performed.

Consistent with prior studies^45,46^, azithromycin (Fig. 3A) required substantial concentration increases to eradicate biofilms (MBEC culture) compared to planktonic MBCs, with *E. coli* and *S. aureus* requiring 4.74-fold (*p* < 0.001; log(BF_10_) = 3.8) and 20.94-fold (*p* = 0.003; log(BF_10_) = 3.0) increases, respectively. Notably, azithromycin MBEC values exceeded peak serum and tissue concentrations achievable with therapeutic dosaging^47^, highlighting its limited utility in eradicating biofilm infections. Encapsulation in nanodroplets (AZ-ND) significantly enhanced azithromycin efficacy, requiring markedly lower concentrations to achieve biofilm matrix dispersal and bacterial eradication, both with and without FUS. Despite the protective effect observed against planktonic species, it is likely that the enhanced penetration of NDs through the biofilm matrix, even in the absence of ultrasound, allow them to access deeper bacterial populations, bypassing the diffusion barriers imposed by the EPS more effectively than free drug formulations. Indeed, this hypothesis is supported by cell uptake studies, as detailed in later sections. Against *E. coli* clinical isolate biofilms, AZ-ND/FUS reduced MBECs relative to free azithromycin by 57.03-fold (*p* = 0.009; log(BF_10_) = 2.2) for biomass removal, 21.1-fold (*p* < 0.001; log(BF_10_) = 7.1) for metabolic inhibition, and 49.52-fold (*p* < 0.001; log(BF_10_) = 5.1) for a 3-log reduction in culturable bacteria. *S. aureus* biofilms showed similar susceptibility patterns, with 36.78-fold (*p* = 0.025; log(BF_10_) = 1.5), 32.06-fold (*p* < 0.001; log(BF_10_) = 6.2), and 78.48-fold (*p* = 0.003; log(BF_10_) = 3.0) reductions in biomass, metabolic, and culture MBEC values, respectively. Remarkably, culturable MBEC values for both species treated with AZ-ND/FUS were statistically insignificant from planktonic MBC levels (*p* = 0.525; log(BF_10_) = −1.0 for *E. coli* and *p* = 0.505; log(BF_10_) = −0.9 for *S. aureus*), underscoring the potent antibiofilm effects of the ND/FUS combination.

Besifloxacin (Fig. 3B) demonstrated similar trends, with 4.45-fold (*p* < 0.001; log(BF_10_) = 4.1) and 7.02-fold (*p* = 0.105; log(BF_10_) = 0.4) higher drug concentrations required to eliminate biofilms of *E. coli* and *S. aureus*, respectively, compared to planktonic cells (MBEC-culture vs. MBC). The antibiofilm efficacy of besifloxacin was notably isolate-dependent in *S. aureus*, where two osteomyelitis strains (BJB2-13, BJB2-23) exhibited heightened resistance to both free drug and BF-ND treatments across biomass, metabolic viability, and culturability measures. This resistance was mitigated with the addition of FUS (BF-ND/FUS), with 42.44-fold (biomass; *p* = 0.073; log(BF_10_) = 0.6), 29.77-fold (metabolic; *p* = 0.059; log(BF_10_) = 0.8), and 30.54-fold (culture; *p* = 0.088; log(BF_10_) = 0.5) reductions in MBEC values across all isolates of *S. aureus* compared to free besifloxacin, with comparable reductions observed in *E. coli*.

Consistent with planktonic results and its mechanism of action, free polymyxin B (Fig. 3C) demonstrated species-specific biofilm responses, requiring 32.71-fold (*p* = 0.003; log(BF_10_) = 2.9) and 186.43-fold (*p* = 0.005; log(BF_10_) = 2.6) higher concentrations for biofilm eradication relative to MBC in *E. coli* and *S. aureus*, respectively. Indeed, the culturability MBEC was so high in *S. aureus* that it precluded the production of PMB-NDs for evaluation, explaining the absence of the data in corresponding analyses. Nonetheless, PMB-NDs combined with FUS achieved 203.18-fold (*p* = 0.110; log(BF_10_) = 0.3) and 44.98-fold (*p* = 0.005; log(BF_10_) = 2.6) reductions in metabolic and culturability MBEC values in *S. aureus*, and smaller yet significant reductions of 5.23-fold (*p* = 0.003; log(BF_10_) = 3.1) and 4.61-fold (*p* = 0.006; log(BF_10_) = 2.5) in *E. coli*. Interestingly, biomass removal was comparable across PMB treatments against *S. aureus* biofilms, with all requiring similar concentrations to eliminate 90% of the matrix.

The ruthenium polypyridyl complex (Fig. 3D) exhibited the lowest MBEC increase relative to MBC (MBEC-culture; 2-fold for *E. coli*, 4.44-fold for *S. aureus*). Ru-NDs combined with FUS further reduced MBEC values for biomass, metabolic, and culture endpoints by an average of 12.30-fold (*p* < 0.001; log(BF_10_) = 9.0) for *E. coli* and 11.43-fold (*p* < 0.001; log(BF_10_) = 6.4) for *S. aureus* relative to free drug, further confirming the synergy of this approach.

Taken together, *E. coli* and *S. aureus* exhibited an average increase in culturable MBEC of 28.5-fold and 38.6-fold, respectively, across all free drugs. ND/FUS treatment enhanced biofilm dispersal and permeabilization, reducing the required antimicrobial concentrations by 92.3-fold (*p* < 0.001, log(BF_10_) = 19.9) and 26.7-fold (*p* < 0.001, log(BF_10_) = 7.3) in *E. coli* and *S. aureus* biofilms, respectively, across all drug-isolate combinations. These reductions were conserved for both metabolic MBEC (33.9-fold, *p* = 0.010, log(BF_10_) = 1.5) and culturable MBEC (44.4-fold, *p* = 0.015, log(BF_10_) = 1.2), demonstrating potent antibiofilm activity across Gram-negative and Gram-positive isolates. Specifically, the consistency of effects across all three assays reinforces the reliability of ND/FUS in eliminating both biofilm matrices and embedded bacteria, emphasizing its therapeutic potential.

Interestingly, the Gram-positive peptidoglycan cell wall likely influenced dispersal outcomes between *E. coli* and *S. aureus* but did not impact antimicrobial activity. Biofilm dispersal correlated with culturability for ND/FUS treatments against both *E. coli* (*R*^2^ = 0.4629) and *S. aureus* biofilms (*R*^2^ = 0.8101), unlike free antibiotics (*R*^2^ = 0.086 and 0.0263, respectively), aligning with previous studies^46,48^ where antibiotics impacted metabolic activity without disrupting the matrix at equal concentrations. These findings underscore the need for assays measuring both biomass and viability to guide treatment and prevent biofilm persistence.

ND/FUS efficacy against persister cells was also evaluated. Persisters, being dormant and resistant to traditional antibiotics, contribute to clinical recurrence by reconstituting infections once the stress is removed^49–51^. Population analysis profiling identified distinct sensitive and persister subpopulations with increasing antibiotic concentration, consistent with literature findings (Fig. S11)^52,53^. To assess complete cell eradication (CFU < 10^1^ cells/mL), disaggregated biofilms were resuscitated on nutrient-rich media for 3 days before plating^54–56^. Free antibiotics required 975.4-fold and 40.6-fold higher concentrations (*p* = 0.011, log(BF_10_) = 1.4) than the MBC and MBEC, respectively, to eliminate persister cells, highlighting the challenge of treating these populations. ND/FUS successfully reduced these concentrations by 25.5-fold (*p* = 0.011, log(BF_10_) = 1.4) compared to free antibiotic, with a particularly marked potentiation effect observed for PMB-NDs against *S. aureus* persisters (91.3-fold reduction; *p* = 0.003, log(BF_10_) = 2.9). Although the potentiation effect was less pronounced for persisters than for planktonic or biofilm-residing cells, ND/FUS showed enhanced efficacy against all biofilm-associated bacteria investigated at clinically feasible antimicrobial concentrations, offering a promising strategy against distinct biofilm defense mechanisms.

### Combatting different clinical biofilm infections

To broaden the scope of the ND/FUS system, its efficacy was evaluated against mature biofilms grown in diverse artificial media under flow conditions to mimic various clinical states (Fig. S12), including cystic fibrosis (CF) sputum (*P. aeruginosa*), chronic wound constituent media (*P. aeruginosa*), prosthetic joint infection synovial fluid (*S. aureus*), and UTI artificial urine (*E. coli*).

**Fig. 4:**
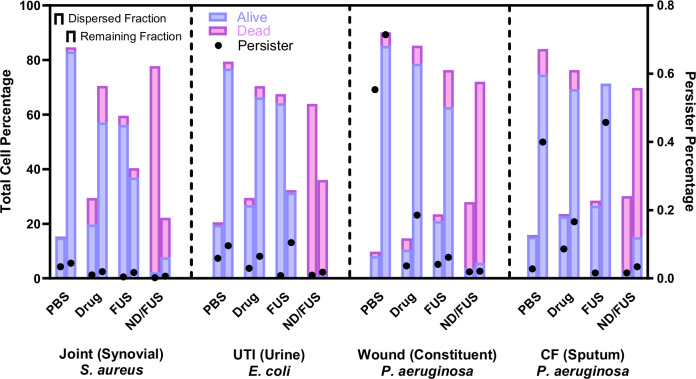
Viability evaluation of *S. aureus, E. coli*, and *P. aeruginosa* biofilms in simulated infection conditions representative of joint infection, UTI, chronic wound/cystic fibrosis, respectively. Treatments included PBS, drug only (besifloxacin), focused ultrasound only (FUS), or besifloxacin-loaded nanodroplets with FUS (ND/FUS) flowed through the biofilm at sub-MBC concentrations. For each treatment, both the fraction of cells dislodged from the biofilm during treatment under flow (dispersed fraction; first bar) and residual biofilm cells (remaining fraction; second bar) were harvested and analyzed using flow cytometry. Charts display the proportion of metabolically viable cells (purple), dead cells (pink), and dormant persister cells (black dot) of all recorded event as stacked bars such that both the stacked bars in the first and second fraction sum to 100%, to represent the total cell percentage. Each section of the figure corresponds to a specific infection model as differentiated by dotted lines: Joint—*S. aureus*; UTI—*E. coli*; Chronic wound—*P. aeruginosa*; Cystic fibrosis—*P. aeruginosa*. All results shown represent three biological replicates.

Viability analyses using RedoxSensor Green and propidium iodide^57^ in flow cytometry demonstrated that ultrasound treatment alone failed to induce substantial cell death in either the fraction of cells dispersed during treatment (dispersed) or the residual adherent biofilm cell population (adherent), relative to untreated flow controls (Fig. 4)^58^. However, ultrasound exposure did promote the resuscitation of dormant persister cells, as identified through selective metabolic and viability gating. Across all tested disease models and strains, the persister cell population decreased from 0.241 ± 0.174% to 0.088 ± 0.051% upon FUS exposure. This phenomenon suggests that ultrasound can potentially enhance nutrient and waste exchange within the biofilm microenvironment, facilitating the reactivation of dormant cells^59^.

FUS treatment alone also triggered the dispersal of biofilm-residing cells, releasing an average of 44.85% of the biofilm population across all tested disease models. The fraction of dispersed cells varied depending on the strain and the disease model. Biofilms grown within chronic wound and CF environments exhibited the greatest resistance to ultrasound-mediated dispersal, with dispersal rates of 23.54% and 28.62%, respectively. This resistance is likely attributable to the alginate EPS matrix produced by *P. aeruginosa*, which reinforces adhesion to the flow cell polymeric substrate.

In contrast, besifloxacin-loaded nanodroplets exposed to FUS (BES-ND/FUS) significantly increased antimicrobial activity on both dispersed and adherent fractions across all disease models, achieving a 90.31% cell kill relative to free drug (12.49%). Moreover, ND/FUS nearly eliminated all persister cells, reducing their population to less than 0.015% of the biofilm-residing cell population, compared to 0.076% induced by free drug alone.

This persister-enriched population was primarily confined to the adherent fraction (0.019%) across all tested treatments, supporting the hypothesis that dormant cells preferentially reside within the deeper layers of the biofilm matrix.

The ability of ND/FUS to eradicate the initially dispersed populations (93.7% cell kill) may alleviate concerns about ultrasound-induced sepsis due to uncontrolled biofilm dispersal^60^. Indeed, these detached cells are thought to express a specialized phenotype that makes them better at forming new biofilms and exhibit increased resistance to antimicrobials compared to both planktonic and resuspended biofilm cells^61,62^. By significantly reducing the viability of these detached clusters, the ND/FUS platform could potentially overcome the limitations of ultrasound-mediated dispersal, preventing recurrent chronic infections and relocation of infections to new sites.

### Subcellular quantification of planktonic drug uptake

To investigate the mechanism of action behind the increased efficacy observed from ND/FUS, the antibiotic internalization in bacterial subcellular compartments was evaluated. As previous developed assays were either too limited in spatial resolution for subcellular compartment analysis^63^ or lacked analytical sensitivity for biofilm quantification^64^, a new protocol based on inductively coupled plasma mass spectrometry (ICP-MS) analysis of europium (LOD = 24.35 ppq) and ruthenium (LOD = 275 ppq) was established^65–69^.

Following conventional chelation procedures, successful metalation and purification of antimicrobials was confirmed through NMR and HPLC-ESI-MS, showing characteristic chemical shifts consistent with lanthanide chelated compounds (Fig. S13). Notably, metalated antimicrobials in solution or loaded onto nanodroplets (Fig. S14) demonstrated no significant decrease in MIC across all isolates, with most exhibiting a decreased inhibitory effect (Fig. S15). Uptake of sub-MIC doses of metalated antimicrobials was evaluated following fractionation of subcellular bacterial compartments^64,70^. Bulk uptake was divided against the volume of each compartment to evaluate the percentage injected dose per compartment volume (% ID/cm^3^; Fig. S18).

**Fig. 5:**
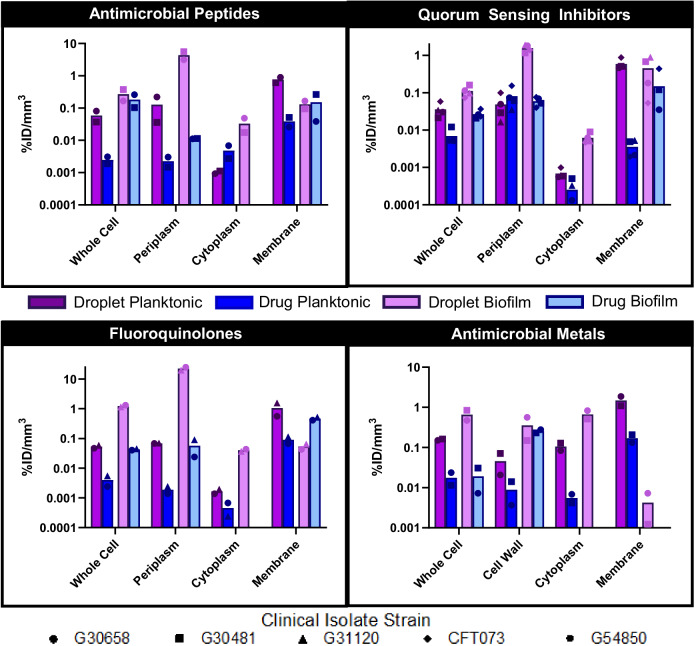
Cellular and subcellular uptake of free and nanodroplet-encapsulated antimicrobials in planktonic and biofilm-embedded *E. coli* clinical isolates, as quantified using ICP-MS. Antimicrobial uptake is expressed as a percentage of the administered dose normalized to the volume of each subcellular compartment. Data points correspond to different bacterial clinical isolates with each data point representing three biological replicates. Bars indicate mean values across tested isolates. Bars not shown for certain subcellular compartments indicate levels of antimicrobial uptake below the detection limit of analysis. The distinct distribution profiles observed, with preferential accumulation of the nanodroplet-encapsulated antimicrobials within the bacterial membranes and cytoplasmic fractions, suggest that the nanoparticulate carrier can overcome the penetration and localization limitations inherent to the free antimicrobial agents.

**Fig. 6:**
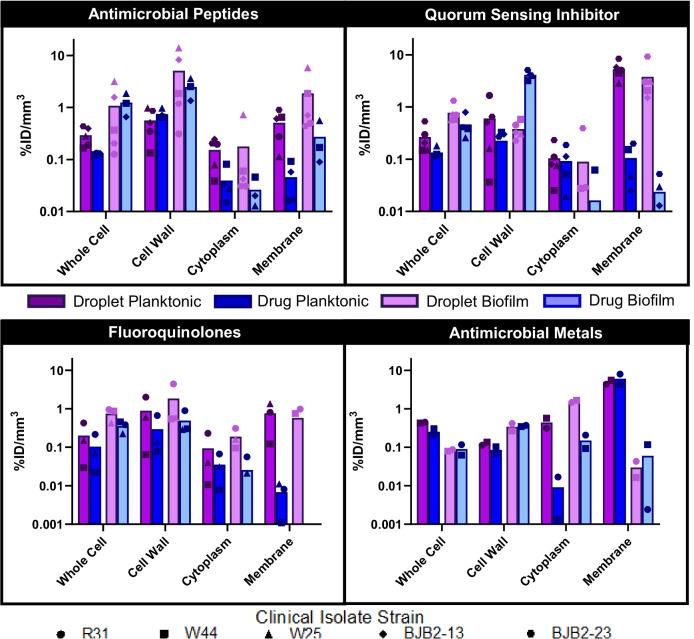
Cellular and subcellular uptake of free and nanodroplet-encapsulated antimicrobials in planktonic and biofilm-embedded *S. aureus* clinical isolates, as quantified using ICP-MS. Antimicrobial uptake is expressed as a percentage of the administered dose normalized to the volume of each subcellular compartment. Data points correspond to different bacterial clinical isolates with each data point representing three biological replicates. Bars indicate mean values across tested isolates. Bars not shown for certain subcellular compartments indicate levels of antimicrobial uptake below the detection limit of analysis. The distinct distribution profiles observed, with preferential accumulation of the nanodroplet-encapsulated antimicrobials within the bacterial membranes and cytoplasmic fractions, suggest that the nanoparticulate carrier can overcome the penetration and localization limitations inherent to the free antimicrobial agents.

Encapsulation of the antimicrobials within the nanodroplet platform increased the bulk planktonic uptake across all drug-isolate combinations (Figs. 5, 6, S16, S17; 2.34-fold; *p* < 0.001, log(BF_10_) = 6.0). Interestingly, the subcellular distribution profiles differed markedly between free drug and their nanodroplet-loaded counterparts. With azithromycin, the free drug predominantly localized within the cell wall, whereas the nanodroplet-encapsulated form localized within the membrane-bound fraction. Across all antimicrobials, drug concentration within membrane-bound fractions was ~3.73-fold higher (*p* = 0.005, log(BF_10_) = 5.2) when delivered via nanodroplets. This preferential membrane localization beyond the peptidoglycan layer, despite the larger size of the nanodroplets relative to small molecule drugs, indicates capacity for focused ultrasound-triggered internalization and drug release. Indeed, the nanodroplet formulations also exhibited slightly elevated (3.64-fold; *p* = 0.022, log(BF_10_) = 1.4) cytoplasmic uptake on average, potentially due to spontaneous vaporization-induced pore formation in the plasma membrane.

In contrast, the ruthenium complex displayed substantial accumulation in the membrane fractions for both the droplet-loaded (3.24% ID/cm^3^) and free drug (3.15% ID/cm^3^) arms. In the cytoplasm, ruthenium-conjugated droplets showed an average of 33.9-fold higher internalization compared to free drug. This divergent subcellular distribution profile is likely attributable to the higher calculated lipophilicity of the ruthenium complex (cLog*P*_*O/W*_ – XLOGP3 = 11.49), facilitating increased partitioning and retention within the bacterial cell membrane fractions, potentially mediated through drug-lipid interactions. Indeed, the inclusion of the ruthenium complex within the hydrophobic core of the nanodroplet platform may have further enhanced their membrane permeability to facilitate higher accumulation in the aqueous cellular compartments. Although observed to a lesser extent with the other less lipophilic antimicrobials, this suggests that nanodroplets may overcome particle size limitations and facilitate cellular internalization and cytoplasmic delivery. Combined with FUS activation, this has the potential to improve intracellular biodistribution and overall efficacy of loaded antimicrobials.

### Subcellular quantification of biofilm drug uptake

In biofilm-associated bacterial populations, a distinct subcellular distribution profile was evident. The overall uptake of antimicrobials within biofilm-dwelling cells appeared diminished relative to their planktonic counterparts, due to the lower cell density in biofilms (Figs. 5, 6, S16, S17). Normalization to the cell population, however, revealed a significant increase in total antimicrobial uptake within biofilm-residing bacteria across all drug-isolate combinations, excluding the ruthenium complex. This enhancement, measured at 3.9-fold for *S. aureus* (*p* = 0.010, log(BF_10_) = 3.3) and 6.7-fold for *E. coli* (*p* = 0.033, log(BF_10_) = 3.2), indicates a substantial improvement in antimicrobial penetration likely facilitated by the prolonged incubation periods in this study. Indeed, antibiotic diffusion through biofilm structures may be delayed or inhibited by transient binding to extracellular matrix constituents^71^, enabling uptake not achievable within shorter timeframes and potentially mitigating drug efflux within planktonic phenotypes. For *S. aureus*, the augmented antimicrobial uptake is primarily attributed to a 5.7-fold increase (*p* = 0.002, log(BF_10_) = 3.2) in accumulation within the cell wall fraction, likely due to the thick peptidoglycan layer limiting further penetration. Conversely, *E. coli* exhibited a 4.6-fold (*p* = 0.009, log(BF_10_) = 2.0) preferential partitioning into membrane-bound compartments, potentially mediated by interactions with the asymmetric lipopolysaccharide (LPS) outer membrane^72,73^. Despite this, no detectable antimicrobial accumulation was observed within the cytoplasmic fractions of *E. coli*, the primary site of action for many of these agents, likely contributing to the reduced efficacy against biofilm-encased bacteria compared to their planktonic counterparts^74^.

Encapsulation of the antimicrobials within nanodroplets offers a promising strategy to enhance accumulation and intracellular distribution within biofilm-encased bacteria. Passive uptake of antimicrobial-loaded nanodroplets showed a 3.3-fold increase in concentration relative to planktonic bacteria (*p* = <0.001, log(BF_10_) = 3.6). Nanodroplet formulations exhibited a distinct subcellular distribution profile compared to free drugs, showing an 88.9-fold enhanced accumulation in the periplasmic space and a 5.1-fold lower concentration in *E. coli* membrane-bound compartments compared to planktonic forms. However, polymyxin B-loaded nanodroplets showed a 2.4-fold enhancement in membrane localization, consistent with their proposed target site of action^75^. Both Gram-positive and Gram-negative biofilm-embedded bacteria displayed a 2.5-fold increase in antimicrobial accumulation within cytoplasmic fractions relative to planktonic when delivered via the nanodroplet platform, which may explain the observed increase in cell kill of antimicrobial-loaded nanodroplets against biofilm-encased bacteria, compared to the protective effect seen in planktonic cultures. Indeed, comparing the nanodroplet-encapsulated antimicrobials to free drug formulations revealed an 11.1-fold (*p* = 0.005, log(BF_10_) = 2.3) higher accumulation within the cytoplasmic compartment of biofilm-residing bacteria, suggesting more effective penetration and intracellular delivery, overcoming the localization limitations observed with free drug.

Mechanistically, the observed antibiofilm efficacy of ND/FUS is likely not solely attributed to the phenotypic change induced by ultrasound-mediated biofilm dispersal, but rather to alteration of antimicrobial subcellular distribution within the biofilm matrix. Nanoparticulate carriers interact uniquely with both the cell wall^76^ and LPS layers^77^, potentially enhancing penetration and destabilization. While the integrity of nanodroplets upon incorporation into bacterial cells remains unclear^78,79^, their presence can significantly alter subcellular distribution profiles of encapsulated antimicrobial agents. Combined with FUS, cavitation of resultant microbubbles can stimulate controlled release of antimicrobial payloads and perturb bacterial phospholipid bilayers to further enhance penetration^80^. Though subcellular uptake following ultrasound activation was not evaluated due to instrumentation sensitivity limitations, the ability of therapeutic ultrasound to facilitate drug delivery through membrane poration, jetting, or acoustic streaming is well documented^81^. While further investigation into the precise mechanisms governing subcellular trafficking of these nanoscale carriers is warranted, the results nonetheless underscore the potential of antimicrobial-loaded nanodroplets to overcome the penetration and localization limitations of antimicrobials within the complex biofilm milieu for effective therapy.

## Outlook

In this study, we describe a stimuli-responsive drug delivery platform for simultaneous biofilm disruption and intracellular drug delivery, significantly reducing the required antimicrobial concentrations for effective treatment and clearance of clinical isolate biofilm infections in vitro. By leveraging nanodroplets, we achieve a significant enhancement in subcellular antimicrobial uptake prior to ultrasound exposure with preferential uptake at the site of antimicrobial action compared to free drug. Subsequent ultrasound exposure triggered the extensive dispersal of the protective biofilm matrix, enabling significant eradication of biofilm-dwelling bacteria—even after resuscitation attempts—at significantly lower drug concentrations compared to free antimicrobials. This is particularly important given that pharmacokinetic analyses suggest that plasma concentrations of most conventional antimicrobial dosages are likely insufficient to eradicate persister cell populations^82^.

Notably, our study utilized a commercial ultrasound array and a formulation based on a commercial contrast agent, paving the way for seamless clinical translation. Moreover, whilst our study utilized existing antimicrobial agents, it could be easily adapted for delivery of novel-antimicrobials/antibiofilm compounds, such as matrix-degrading enzymes or bioactive gases, to further mitigate the risk of resistance development. The demand for innovative solutions is pressing, and by harnessing the synergistic effects of ultrasound-mediated biofilm disruption and targeted antimicrobial delivery, ND/FUS offers a promising new approach for the management of chronic, biofilm-associated infections – a growing concern accelerated by the alarming rise of antimicrobial resistance^83^.

## Methods

### Materials

All lipids were purchased from Avanti Polar Lipids (Alabaster, AL). All chemicals and solvents were purchased from Sigma Aldrich (Gillingham, UK) unless otherwise noted.

### Synthesis and characterization of ruthenium polypyridyl azide complex

Ruthenium polypyridyl azide complex was synthesized using modified protocols reported previously^18,84^. Briefly, an equimolar suspension of dichloro(1,5-cyclooctadiene)ruthenium(II) chloride ([Ru(COD)Cl_2_]_n_) and 4,4’-di-*tert*-butyl-2,2’-dipyridyl (BBBPY) in dimethylformamide (DMF) was refluxed for 4 h at 140 °C in the dark under an inert N_2_ atmosphere. The solvent was immediately removed using a rotary evaporator and the obtained solid was washed with diethyl ether and dried. The resultant compound was then reacted with equimolar 4,4’-bis(bromomethyl)-2,2’-bipyridine (AA Blocks, San Diego, CA) in a suspension of 2:1 v/v ethanol/water and heated to 90 °C under reflux for 10 h. After cooling the reaction to room temperature, the solvent was evaporated using a rotary evaporator. The resultant solid was dissolved in deionized water and filtered with a 5.0 µm glass fiber (GF) membrane filter. A precipitate was formed by adding a saturated aqueous solution of ammonium hexafluorophosphate before washing with water and diethyl ether. Ten times molar excess sodium azide was added to a solution of resultant compound in acetonitrile and stirred at room temperature for 5 days before filtering to remove precipitated NaBr. The solution was then precipitated using a saturated aqueous solution of ammonium hexafluorophosphate before washing with deionized water and drying under rotary evaporation. The resultant solid was then reacted with 1,2-distearoyl-sn-glycero-3-phosphoethanolamine-n-[dibenzocycooctyl(polyethylene glycol)-5000] (DSPE-PEG(5000) DBCO) in 10 vol% acetonitrile and 90 vol% chloroform overnight at 37 °C under 200 rpm mixing.

The identity and purity of the ruthenium polypyridyl azide complex was confirmed by nuclear magnetic resonance (NMR) and high-performance liquid chromatography electrospray ion source mass spectrometry (HPLC-ESI-MS). NMR spectra were acquired on a Bruker Ascend 400 MHz spectrometer with 30° read pulses and a 4-second relaxation delay on a spectral width of 8.000 Hz at 298 K and processed using MestreNova 14.3.1. HPLC-MS was performed using an Agilent 1260 Infinity II (Agilent, Santa Clara, CA) with an Agilent 1290 Infinity II flexible pump (G7104A), a 1260 Infinity II photodiode array detector (G7117C), a 1260 Infinity II fluorescence detector spectra (G7121B), a Infinity lab liquid chromatography mass detector (LC/MSD; G6125B), and a BEH C4 300 Å 3.5 µm column (Waters, Milford, MA). HPLC was conducted using 0.1% trifluoroacetic acid in water (A) and acetonitrile (B) with a linear gradient starting at 70% A + 30% B to 0% A + 100% B over 5 min and maintained for a following 3 min. Column temperature and flow rate were maintained at 60 °C and 0.7 mL/min, respectively, throughout. Absorbance and fluorescence spectra of all compounds were collected using UV compatible microplates (UV-Star µClear, Grenier Bio-One; Stonehouse, UK), scanning from 220 nm to 1000 nm following blank correction for the former, and excitation wavelength of 435 nm and emission wavelengths of 485 nm to 740 nm, for the latter. Extinction coefficient values were determined spectroscopically using the Beer-Lambert equation, using experimentally derived pathlength correction values of each well plate from absorbance properties of water at 900 nm and 977 nm.

### Formation and characterization of europium-chelated antimicrobial complexes

Metalation of europium-azithromycin (Eu-AZM) and europium-besifloxacin (Eu-BES) were performed using similar methods as those previously reported for multivalent metals^65,85,86^. Briefly, an equimolar (1 mM) solution of antimicrobial and potassium hydroxide was dissolved in a hot methanolic solution before 0.33 mM europium chloride (EuCl_3_) was added. The reaction was performed under reflux at 60 °C for 3 h before filtering using a 3.0 µm GF membrane filter. The solution was immediately placed in ice for 2 h to instigate crystallization before drying under centrifugal vacuum evaporation. Metalation of europium-polymyxin B (Eu-PMB) was performed as previously described in patent^87^. Briefly, an equimolar (0.05 mM) solution of Polymyxin B pentasulfate and europium chloride (EuCl_3_) was dissolved in 0.05 M ammonium acetate buffer (pH = 5.5) and incubated at room temperature briefly. The mixture was then filtered using a Sephadex G-25 column and concentrated under centrifugal vacuum evaporation to remove the volatile buffer.

The identity and purity of europium-chelated antimicrobial complexes were confirmed by NMR and HPLC. NMR spectra were acquired on a Bruker Ascend 400 MHz spectrometer with 30° read pulses and a 4-second relaxation delay on a spectral width of 8.000 Hz at 298 K and processed using MestreNova 14.3.1. Chemical shift (δ) values are given in parts per million and are referenced to residual solvent unless otherwise stated; *J* values are quoted in Hz. HPLC was performed using an Agilent 1260 Infinity II (Agilent, Santa Clara, CA) with an Agilent 1290 Infinity II flexible pump (G7104A), a 1260 Infinity II photodiode array detector (G7117C), 1260 Infinity II fluorescence detector spectra (G7121B), Infinity lab liquid chromatography mass detector (LC/MSD; G6125B), and a BEH C4 300 Å 3.5 µm column (Waters, Milford, MA). HPLC-UV, FL, and MS for azithromycin samples were conducted using 90% methanol (A) and 10% 0.02 M potassium phosphate dibasic buffer in water with 2 mL/L triethylamine (B). Column temperature and flow rate were maintained at 40 °C and 1.0 mL/min, respectively, throughout. UV and fluorescence detection were performed at 210 nm and 315 nm, respectively. Besifloxacin samples were conducted using 20 vol% acetonitrile: 80 vol% methanol (A) and 0.1% trifluoroacetic acid in water (B) with a gradient elution of 20% A + 80% B for 20 min, increasing to 50% A + 50% B over 15 min, returning to 20% A + 80% B over 1 min, and maintained for 1 min. Column temperature and flow rate were maintained at 30 °C and 1.0 mL/min, respectively, throughout. UV detection was performed at 290 nm. Polymyxin B samples were conducted using acetonitrile (A) and 0.03 M anhydrous sodium sulfate in water, adjusted to pH 2.3 with dilute phosphoric acid (B), at a 20% A + 80% B 1.0 mL/min flow. UV detection was performed at 215 nm at 30 °C. Absorbance spectra of all compounds were collected using UV compatible microplates (UV-Star µClear, Grenier Bio-One; Stonehouse, UK), scanning from 220 nm to 1000 nm following blank correction. Extinction coefficient values were determined spectroscopically using the Beer-Lambert equation, using experimentally derived pathlength correction values of each well plate from absorbance properties of water at 900 nm and 977 nm.

### Antimicrobial-loaded nanodroplet preparation

To form drug-loaded nanodroplets, a protocol was adapted from patent information of the clinical microsphere agents, Definity™ and Definity RT™ (Lantheus Medical Imaging, N. Billerica, MA)^11^. Briefly, a 6.66 × 10^–7 ^mol lipid mixture consisting of 81 mol% 1,2-distearoyl-sn-glycero-3-phosphocholine (DSPC) in a mixture of 90 vol% chloroform and 10 vol% methanol, 10 mol% 1,2-distearoyl-sn-glycero-3-phosphate (DSPA) in a mixture of 65 vol% chloroform, 35 vol% methanol, and 8 vol% MilliQ water, and 8 mol% 1,2-distearoyl-sn-glycero-3-phosphoethanolamine-N-[methoxy(polyethylene glycol)-5000 (DSPE-mPEG-5000) in a mixture of 90 vol% chloroform and 10 vol% methanol, were combined in 0.5 dram borosilicate glass vials (Ampulla, Hyde, UK). In formulations containing antimicrobials, the agent was added to the vial prior to the addition of the lipid mixture. Each drug was dissolved in either a 90 vol% chloroform and 10 vol% dimethyl sulfoxide (AZM and PMB); 10 vol% benzyl alcohol, 10% dimethyl sulfoxide and 80% chloroform (BES); or 5 vol% acetonitrile and 95 vol% chloroform (ruthenium). In all cases, europium-metalated antimicrobials were dissolved in the same solvents as their unmetalated counterparts under 2000 rpm agitation at 60 °C for 1 h to ensure dissolution. In case of ruthenium microbubble preparation, DSPE-mPEG-5000 was replaced with the synthesized ruthenium polypyridyl azide complex conjugated to DSPE-mPEG(5000) DBCO within the lipid mixture. Lipid solutions were slowly vortexed, dried under low-pressure nitrogen gas, and placed under vacuum to form a lipid film. Films were rehydrated with 1 mL 80:10:10 v/v acetate buffer (pH = 5.2; 0.074 mg anhydrous sodium acetate and 0.006 mg glacial acetic acid in MilliQ), propylene glycol, and glycerol. Vials were heated at 60 °C using a water bath for 1 min, and sonicated (QSonica Q125, probe diameter 2 mm, 125 W, 20% intensity) using a probe sonicator for 3 min (30 s cycles × 6) to suspend the lipid film. The headspace within the vial was replaced with a 1:1 mixture of perfluoropropane (C_3_F_8_; PFP) and perfluorobutane gas (C_4_F_10_; PFB) before mechanical agitation for 45 s using a VialMix® shaker (Lantheus Medical Imaging, N. Billerica, MA) or similar. Samples were cooled before use and were measured immediately to assess precursor microbubble size and concentration whilst mitigating temporal effects. The vials were then cooled in 30 vol% propylene glycol in water at −10 °C under N_2_ pressure until the solution became clear, representing the condensation of the precursor microbubble to a nanoformulation state.

### Nanodroplet characterization

Size and concentration of nanodroplets and precursor microbubbles were determined using simultaneous interferometry (Videodrop; Meritics, Bedfordshire, UK), dynamic light scattering (DLS; Zetasizer Nano ZS; Malvern, Malvern, UK), and electro-impedance volumetric zone sensing (Coulter Counter Multisizer Z3; Beckman Coulter, Brea, CA). Coulter measurements were conducted using samples diluted in Isoton-I electrolyte solution (Beckman Coulter, Brea, CA), where a background count of buffer was taken and subtracted from the final count. Number and size distributions were measured using a 20-µm aperture, detecting diameters from 0.4 to 12 µm. Both interferometry and DLS measurements were conducted using samples diluted in deionized water. For the former, 5 × 60 s videos were collected and analyzed using the manufacturer’s built-in software.

Zeta potential of nanodroplets was determined using dynamic light scattering (DLS; Zetasizer Nano ZS; Malvern, Malvern, UK), using samples diluted in 10 mM phosphate buffer (pH = 7.0), 10 mM carbonate/bicarbonate buffer (pH = 7.0), and 10 mM ammonium acetate buffer (pH = 7.0). Measurements were performed using the Smoluchowski protocol for up to 100 runs, with three measurements conducted per sample.

### Antimicrobial loading optimization

To determine the most advantageous concentration of antimicrobial loaded within the nanodroplet formulation, systematic drug loading was conducted by adding 0–50 mol% antimicrobial within the base formulation detailed above. Nanodroplets were prepared using the mechanical agitation method described, and analyzed using electro-impedance volumetric zone sensing, DLS, and interferometry. Optimal antimicrobial loading was determined as a basis of concentration above 10^11^ particles/mL, size below 200 nm, <1% particles above 0.8 µm, encapsulation efficiency above 70%, and serum half-life beyond 10 h.

### Ultrasound setup

A custom-built ultrasound setup was designed for these experiments, combining a clinical ultrasound array, high-speed optical imaging, and fluorescence imaging. Briefly, a C5-2 broadband 128-element curved array transducer (Philips, Guildford, UK) was programmed using the Verasonics Vantage 256 ultrasonic research system (Verasonics, Kirkland, WA). The transducer was driven at its center frequency, 3.125 MHz, with a frequency bandwidth of 1/3, at a focal point of [−3.8 mm, 0.2 mm, 190 mm], with a right-angle polished stainless steel acoustic mirror to reflect the acoustic signal. For vaporization, the arbitrary transmit waveform comprised of a 99.0% active transmit driver during the half cycle period, 20 half-cycle bursts, and positive polarity during the first half-cycle with equalization pulses added to the start and the end of the burst. For delivery of ultrasound, the arbitrary transmit waveform comprised of two alternating waveforms, each with half apodization, consisting of 99.0% active transmit drivers during the half cycle period, 1000 pulses of 156 half-cycles (effective “on” of 25 µs/pulse), 1% duty cycle, and 332.9 kPa pnp. Acoustic pressure calibrations and field characterization were performed using a needle hydrophone (PA3382; Precision Acoustics, Dorchester, UK) on a micropositioner setup (Newport; Oxford, UK) connected to a 600 MHz oscilloscope (Teledyne Le Croy; Chestnut Ridge, NY). Detailed ultrasound parameters and scripts can be found in the Vantage Sequence code in Appendix A in the Supplementary Information file.

A 20X objective lens with a numerical aperture of 0.5 and working distance of 3.5 mm (UMPLFLN20XW, Olympus; Tokyo, Japan) was focused on the midplane of a 200 µL channel volume (800 µm channel height, 2.5 cm^2^ growth area) flow cell (µ-Slide I Luer, Ibidi GmbH; Gräfelfing, Germany) and coupled to both a high-speed camera (HPV-X2, Shimadzu; Tokyo, Japan) and CMOS by way of dichroic mirrors and a 4x and 0.5x intermediate lens (WI-DPMC, Olympus; Tokyo, Japan), respectively. A 635 nm high-speed laser illumination system (Cavilux; Cavitar, Tampere, Finland) with 10 ns pulse duration through a 1.4 NA achromatic/aplanatic condenser (U-AAC, Olympus; Tokyo, Japan) was triggered using the output from the Verasonics system, in-line with the ultrasound pulse. Epifluorescent illumination was provided by a 445 nm high-intensity LED light source (SOLIS-1 C, Thorlabs, Ely, UK). High-speed images were acquired using a FTCMOS2 image sensor at 10 million frames per second (Mfps) for 128 frames, with a 50 ns exposure time.

### Acoustic characterization

To determine acoustic vaporization thresholds, high-speed imaging was used in-line with methods previously reported^25^. Briefly, vaporization was defined as the appearance in gray-scale contrast in the optical focal region above that of noise, as calculated by particle counts during exposure compared with before ultrasound exposure. Complete vaporization was defined as the appearance in gray-scale contrast at 3.6 MPa at 60 °C. As acoustic pressures were increased stepwise, any statistically significant increase in contrast compared to the previous pressure was defined as an increase in acoustic vaporization. For each acoustic pressure, nanodroplets were flowed through the flow cell using a syringe pump at a constant rate of 0.36 mL/h.

### Nanodroplet stability

The stability of formulated nanodroplets was evaluated via size and concentration measurements, and ultrasound acoustic responsiveness. In all cases, particle suspensions were diluted into either PBS or 50 vol% PBS/50 vol% fetal bovine serum (FBS) at 1 × 10^9^ nanodroplets/mL and kept at 37 °C to represent in vivo conditions. Particle suspensions were removed at 10, 24, 50, and 100 h for size and concentration analysis using electro-impedance volumetric zone sensing, DLS, and interferometry. Spontaneous vaporization was defined as the percentage of particles detected above 0.8 µm. Samples were stored at room temperature and removed at 15, 30, 45, 60, 75, 90, 105, and 120 days for analysis of storage stability as before.

### Clinical isolate identification and sequencing

*S. aureus* and *E. coli* clinical isolates were identified, obtained and sequenced from previous studies conducted across three UK hospital trusts as part of the UK Clinical Infection Research Group (UKCIRG): Oxford University Hospitals NHS Trust (Oxford, UK), Brighton and Sussex University Hospitals NHS Trust (Brighton, UK), and University Hospitals Plymouth NHS Trust (Plymouth, UK).

### Artificial bacterial culture medium

To better represent the disease microenvironment of various biofilm-related diseases, four artificial bacterial culture media were utilized. Composite synthetic human urine was prepared per Ipe et al. to match physiological ranges of key ions and proteins in human urine, with the addition of 5 mg/mL human plasma fibrinogen to facilitate biofilm attachment^88^. To mimic prosthetic joint infections and osteomyelitis, artificial synovial fluid was prepared using biochemical compositions outlined by Stamm et al.^89^. Similarly, artificial sputum medium was prepared concordant with Kirchner et al.^90^ to mimic *P. aeruginosa* growth within the CF microenvironment. Artificial wound constituent media was prepared following LuTheryn et al.^91^ to reflect the pathophysiological microenvironment of biofilms found within a chronic wound. Growth kinetics were performed for all artificial culture media.

### Planktonic antimicrobial activity

The antimicrobial inhibitory properties of the nanodroplet platform were assayed using the broth microdilution reference method per ISO 20776-1 recommendation. Briefly, *S. aureus* and *E. coli* isolates were streaked, cultured, and diluted in un-supplemented Mueller-Hinton broth to a final inoculum of 5 × 10^5^ CFU/mL, as determined by optical density calibrations. Bacterial suspensions were treated at different antimicrobial molar concentrations either with free drug, drug-loaded nanodroplets alone, or drug-loaded nanodroplets exposed to ultrasound. Microplates were incubated at 37 °C for 20 h before visual confirmation of minimum inhibitory concentrations (MIC). As defined by EUCAST, MIC was read as the lowest concentration of antimicrobial agent that completely inhibited the growth of the organism, by way of turbidity, as detected by the unaided eye. Metabolic inhibitory measurements were conducted following growth in either brain heart infusion (*S. aureus*) or synthetic human urine (*E. coli*) using 5% v/v alamarBlue™ (resazurin). As prior, initial inoculi of 5 × 10^5^ CFU/mL, as determined by optical density calibrations, were treated with equivalent drug molar concentrations and diluted serially with PBS prior to incubation at 37 °C for 20 h. Resazurin was then added and incubated for 1 h, and measured on fluorescence (excitation 540 nm, emission 560–620 nm). Wells on each row with culture medium without cells were used as negative sterility controls, and wells with inoculum and no antimicrobial were used as growth controls. Each drug-isolate combination concentration gradient was repeated in duplicate. The calculation of the concentration required to inhibit the net increase in metabolic viability by 50% was calculated from a dose response curve by nonlinear regression, and are expressed as mean values ± SEM. As all dose response curves produced a biphasic or triphasic response due to the presence of dormant persister cells, only the first curve, corresponding to metabolically active cells, was taken into consideration for calculations.

Bactericidal efficacy was assessed using agar plate microdilution subculture of stationary phase (OD = 0.8) *S. aureus* (4.74 × 10^8^ CFU/mL) and *E. coli* (3.84 × 10^8^ CFU/mL) in brain heart infusion and synthetic human urine, respectively. Bacterial cultures were treated at different antimicrobial molar concentrations either with free drug or drug-loaded nanodroplets for a 24-h incubation period before ultrasound exposure, if applicable, was performed using previously mentioned parameters. Following ultrasound exposure, bacterial suspensions were incubated for a further 3 h to allow any relevant antimicrobial mechanisms of action to occur. Bacterial suspensions were then centrifuged at 5000 RCF for 10 min to remove the remaining drug before resuspending in PBS and minimize antibiotic carryover^92^. Samples from wells were serially diluted and individually plated on 1.5% w/v agar Luria-Bertani (LB) under standardized conditions as described in document M26-A^93^. The minimum bactericidal concentration (MBC) was defined as the concentration required to kill 99.9% of counted colonies relative to the untreated negative growth control following a 24-h incubation period, as calculated from a dose response curve by nonlinear regression. Each sub-culture was plated in triplicate as a technical replicate and each drug-isolate combination concentration gradient was repeated in duplicate as a biological replicate. As before, dose response curves exhibited a multiphasic response, and in some cases a paradoxical effect phenomena^94^, necessitating that only the first curve, corresponding to metabolically active cells, was taken into consideration for calculations.

### Antibiofilm activity

Biofilms were grown using an interlaboratory validated modified Calgary Biofilm Device^95^ (ThermoFisher; Waltham, MA) or a similar custom-peg lid design in the case of ultrasound-exposed biofilms. Both allowed for biofilm growth on removable polypropylene pegs with a high surface area to volume ratio with similar growth kinetics. Both *S. aureus* and *E. coli* biofilms were grown using a starting inoculum of 6.1 × 10^6^ CFU/mL and 7.96 × 10^6^ CFU/mL (OD = 0.01) in brain heart infusion and synthetic human urine, respectively, before incubating at 37 °C for 72 h, with media changes every 24 h. Biofilms were treated at different antimicrobial molar concentrations either with free drug or drug-loaded nanodroplets for a 24-h incubation period before ultrasound exposure, if applicable, was performed using previously mentioned parameters. Following ultrasound exposure, bacterial suspensions were incubated for a further 3 h to allow any relevant antimicrobial mechanisms of action to occur. For biomass experiments, pegs were air dried for 30 min before being transferred to a 0.1% safranin solution and stained for 10 min. Pegs were subsequently removed and washed twice by submerging in deionized water to remove unbound stain before once again air drying for a further 30 min. Safranin was removed by solubilizing pegs in 33% acetic acid for 30 min and measured on absorbance (520 nm).

For metabolic viability and culturability assays, pegs were air dried for 30 min before being solubilized in 4 °C 5 mM ethylenediaminetetraacetic acid in deionized water for 30 min to stimulate nutrient deprivation and biofilm dispersal. Biofilms were then further dislodged by scraping before adding 5% v/v alamarBlue™ (resazurin). Stained bacterial suspensions were incubated for 2.5 h before measuring on fluorescence in triplicate (excitation 540 nm, emission 560–620 nm). Each aliquot was then plated on 1.5% w/v agar LB and incubated for 24 h. As before, the MBEC was defined as the concentration required to kill 99.9% of counted colonies or reduce resazurin signal by 80% relative to the untreated negative growth control following a 24-h incubation period, as calculated from a dose response curve by nonlinear regression. Each drug-isolate combination concentration gradient was repeated in triplicate as a biological replicate.

Persister elimination assays were treated and dispersed in an analogous manner to above. Following biofilm dispersal, bacterial suspensions were resuspended in media and incubated at 37 °C for 72 h to encourage persister resuscitation^96^. Resultant suspensions were then plated on 1.5% w/v agar LB and incubated for 24 h as before. Persister cell elimination was defined as the concentration required to achieve complete eradication of counted colonies below 10° CFU/mL. Each drug-isolate combination concentration gradient was repeated in triplicate as a biological replicate.

### Clinical indication validation

Biofilms were grown using a modified µ-slide I Luer flow cell (50 mm channel length, 5 mm channel width; Ibidi GMBH; Gräfelfing, Germany) whereby outer slide dimensions were cut to the channel width to reduce acoustic attenuation through the otherwise absorbent material. The flow cell was inoculated with OD = 0.01 at the logarithmic stage of growth of *S. aureus*, *E. coli*, or *P. aeruginosa* in either artificial synovial fluid, synthetic human urine, artificial sputum, or wound constituent medium. The flow cell was incubated at 37 °C for 72 h and fresh medium was flowed through daily at 0.6 mL/h for one hour. Confirmation of successful biofilm formation was done under brightfield microscopy and verification of this procedure was performed on select samples using safranin staining.

At the end of the 72-h growth period, the flow cell system was flushed gently with PBS to remove remaining planktonic cells and was placed within a custom-built sample holder inside the custom-designed ultrasound setup. PBS, free besifloxacin, or besifloxacin-loaded nanodroplets were diluted with PBS to an equivalent concentration of 10 µM and flowed into the system at 3.6 mL/h for 15 min, collecting the outflow. In experiments involving ultrasound, samples were exposed using the treatment scheme defined previously, 24 h following inflow. 4 h following treatment, flow cells were flushed with 5 mM EDTA, rapidly pushing back and forth, to dissociate any remnant cells attached to the polymer surface. Flow cells were manually opened and scraped to dislodge the remaining cells. Cell suspensions were washed and pelleted through centrifugation at 5000 RCF for 10 min at 4 °C.

To assess metabolic viability, the *Bac*Light™ RedoxSensor™ Green reagent (ThermoFisher, Waltham, MA) and PI were used per manufacturer instructions, using sodium azide and carbonyl cyanide chlorophenylhydrazone (CCCP) as positive control reagents for Gram-negative and Gram-positive strains, respectively. After staining, both initial outflow and remaining biofilm samples were fixed using 4% paraformaldehyde (Biolegend, San Diego, CA) per manufacturer instructions, and analyzed on the BD LSRFortessa™ flow cytometer for 60 s at “low” (12 ± 3 µL/min). Bacterial populations/milliliter were defined as the number of events recorded within the FSC/SSC preset gate and from the volume aspirated by the flow cytometer over the run time. Bacterial populations were further verified by assessing the preset PI gate, set to evaluate membrane permeable dead cells. Ultrasound-mediated membrane permeability was mitigated by waiting 4 h post-treatment to allow for transient poration or other structural perturbation to recover. Metabolically active cells were defined as the number of events recorded within a “positive” RedoxSensor Green gate from the bacterial populations identified. Dead and persister cells were defined as the number of events recorded within a “negative” RedoxSensor Green gate from the bacterial populations identified. This was further segregated by defining dead cells to be the number of events recorded with both a “very negative” RedoxSensor Green gate and a “strong positive” PI gate; likewise, persister cells were defined as the number of events recorded with a “strong negative” RedoxSensor Green gate and a “strong negative” DAPI gate. Controls for persister populations were generated through 100x MIC ampicillin. To confirm results outputted through FACS, culturability assays were further performed on both the initial outflow and remaining biofilm samples by plating an aliquot of cell suspension after washing on 1.5% w/v agar LB and incubating for 24 h.

### Cell uptake

*S. aureus* and *E. coli* isolates were streaked, cultured, and diluted in either brain heart infusion or synthetic human urine, respectively, in stationary phase to a final inoculum of 1 × 10^8^ CFU/mL. Free drug or antimicrobial-loaded nanodroplets at 0.50 µM were pipetted into each inoculum suspension and incubated at 37 °C for 24 h. Cell suspensions were then washed by centrifugation at 5000 RCF, 4 °C for 10 min. Cell pellets were resuspended in 1 mL PBS before 50 µL was removed for analysis as the whole cell fraction. The remaining fraction was pelleted once more before being resuspended in buffer solution. For *E. coli*, cells were first incubated in a concentrated EDTA-sucrose solution (4 °C, 950 µL buffer with 100 mM Tris-acetate, 500 mM sucrose, 5 mM ethylenediaminetetraacetic acid, 1 mM MgCl_2_ in deionized water) to destabilize the outer membrane before adding 50 µL of 2 mg/mL lysozyme in TE buffer to cleave the periplasmic peptidoglycan layer. This was incubated for 5 min at 37 °C before a final mild osmotic shock (20 µL of 1 M MgSO_4_) was performed to finalize peptidoglycan hydrolysis. For *S. aureus*, cells were instead incubated in a Tris-Sucrose-Magnesium chloride (TSM) buffer (10 mM MgCl_2_, 500 mM Sucrose in 50 mM Tris, pH 7.5) with 0.2 µg/mL lysostaphin and 1x Protease inhibitor for 30 min at 37 °C to isolate the cell wall fraction. In both cases, the suspension was then centrifuged at 3200 RCF for 10 min at 4 °C, collecting the supernatant as either the periplasmic or cell wall fraction for analysis.

The remaining cell pellet was re-suspended in 4 °C 1 mL lysis buffer, 2 µL/mL DNase, and 2 µL 0.5 M EDTA, incubating under agitation at 300 rpm for 15 min. Cell suspensions then underwent three cycles of freeze-thaw, going between −70 °C and 37 °C, with a minimum of 1 h at each temperature. To confirm successful cell lysis through this procedure, brightfield microscopy images were taken on select samples. Samples were then pelleted at 16000 RCF for 72 h, to maintain equivalence with higher G-force rates at lower times, at 4 °C before removing the supernatant (cytoplasmic/protoplast fraction) and pellet (membrane bound fraction) for analysis.

All samples were concentrated using a SpeedVac and digested in 65% w/v nitric acid for a minimum of 24 h, of which 2 h were spent under 60 °C heat. Solution samples were then diluted in MilliQ water to a final nitric acid concentration of 5% w/v, such that observed values were within the range of sensitivity of the machine. Diluted samples were passed through a 0.45 µm polytetrafluoroethylene membrane filter. A calibration curve for both elemental europium and ruthenium generated from certified reference materials was used to quantify the concentration of metal in each sample. To back-calculate for administered dose, a further 10 µL sample of the administered agent was also digested and diluted under similar protocols. All reported uptake concentrations were calculated based on the measured administered dose as assessed through a NexION 5000 ICP-MS (PerkinElmer) with autosampler. Sample solution was mixed with carrier (5% v/v HNO_3_) and internal standard (In, 1 ng/g) before aspiration. Quality control standards were performed every 20 samples and monitored for deviation.

### Statistical methods

All results are analyzed using JASP (v.0.18.3; University of Amsterdam, NL) and expressed as means unless noted otherwise. Where hypotheses were tested by comparing means of two uncorrelated groups, Student’s *t* test (unpaired, two-tailed) was employed. Where correlated, Student’s *t* test (paired) and Bayesian *t* tests (paired) were employed and checked for Bayes factor robustness. Correlation bivariate analyses were conducted using Pearson correlation coefficients.

## Supplementary information

supplementary video 1

supplementary video 2

Supporting Information

## Data Availability

The raw data from which the material presented in this paper is derived are available from the Oxford Research Archive (https://ora.ox.ac.uk/). They may also be obtained from the corresponding author, ES, upon reasonable request.

## References

[1] Costerton, W. et al. The application of biofilm science to the study and control of chronic bacterial infections. *J. Clin. Investig.* **112**, 1466–1477 (2003). 14617746 10.1172/JCI20365 PMC259139

[2] Costerton, J. W., Stewart, P. S. & Greenberg, E. P. Bacterial biofilms: a common cause of persistent infections. *Science* **284**, 1318–1322 (1999). 10334980 10.1126/science.284.5418.1318

[3] Choi, V., Rohn, J. L., Stoodley, P., Carugo, D. & Stride, E. Drug delivery strategies for antibiofilm therapy. *Nat. Rev. Microbiol.* **21**, 555–572 (2023). 37258686 10.1038/s41579-023-00905-2

[4] Akinbobola, A. B., Amaeze, N. J., Mackay, W. G., Ramage, G. & Williams, C. Secondary biofilms’ could cause failure of peracetic acid high-level disinfection of endoscopes. *J. Hosp. Infect.* **107**, 67–75 (2021). 33098959 10.1016/j.jhin.2020.09.028

[5] Claesson-Welsh, L., Dejana, E. & McDonald, D. M. Permeability of the endothelial barrier: identifying and reconciling controversies. *Trends Mol. Med.* **27**, 314–331 (2021). 33309601 10.1016/j.molmed.2020.11.006 PMC8005435

[6] Erriu, M. et al. Microbial biofilm modulation by ultrasound: current concepts and controversies. *Ultrason. Sonochem.* **21**, 15–22 (2014). 23751458 10.1016/j.ultsonch.2013.05.011

[7] Rediske, A. M., Rapoport, N. & Pitt, W. G. Reducing bacterial resistance to antibiotics with ultrasound. *Lett. Appl. Microbiol.* **28**, 81–84 (1999). 10030038 10.1046/j.1365-2672.1999.00461.x

[8] Guo, H. et al. Stimulated phase-shift acoustic nanodroplets enhance vancomycin efficacy against methicillin-resistant Staphylococcus aureus biofilms. *Int J. Nanomed.* **12**, 4679–4690 (2017). 10.2147/IJN.S134525 PMC5501628 28721044

[9] Papadopoulou, V. et al. Overcoming biological barriers to improve treatment of a Staphylococcus aureus wound infection. *Cell Chem. Biol.* **30**, 513–526 (2023). 37148883 10.1016/j.chembiol.2023.04.009 PMC10198964

[10] Durham, P. G. et al. Harnessing ultrasound-stimulated phase change contrast agents to improve antibiotic efficacy against methicillin-resistant Staphylococcus aureus biofilms. *Biofilm* **3**, 100049 (2021). 34124645 10.1016/j.bioflm.2021.100049 PMC8173270

[11] Robinson Simon, P., Siegler Robert, W., Onthank David, C. & Nguyen Nhung, T. Lipid-encapsulated gas microsphere compositions and related methods. US patent US 10022460 B2 (2018).

[12] Gold, K., Slay, B., Knackstedt, M. & Gaharwar, A. K. Antimicrobial activity of metal and metal-oxide based nanoparticles. *Adv. Ther.* **1**, 10.1002/adtp.201700033 (2018).

[13] Nalca, Y. et al. Quorum-sensing antagonistic activities of azithromycin in Pseudomonas aeruginosa PAO1: a global approach. *Antimicrob. Agents Chemother.* **50**, 1680–1688 (2006). 16641435 10.1128/AAC.50.5.1680-1688.2006 PMC1472232

[14] Pompilio, A. et al. Subinhibitory concentrations of moxifloxacin decrease adhesion and biofilm formation of Stenotrophomonas maltophilia from cystic fibrosis. *J. Med. Microbiol.* **59**, 76–81 (2010). 19762476 10.1099/jmm.0.011981-0

[15] Yasir, M., Willcox, M. D. P. & Dutta, D. Action of antimicrobial peptides against bacterial biofilms. *Materials* **11**, 10.3390/ma11122468 (2018). 10.3390/ma11122468 PMC6317029 30563067

[16] Li, F., Collins, J. G. & Keene, F. R. Ruthenium complexes as antimicrobial agents. *Chem. Soc. Rev.* **44**, 2529–2542 (2015). 25724019 10.1039/c4cs00343h

[17] Rutherford, T. J. & Keene, F. R. Stereochemical Control of Donor and Acceptor Groups in a Monomeric Chromophore−Quencher Complex of Ruthenium(II). *Inorg. Chem.* **36**, 2872–2878 (1997). 11669925 10.1021/ic9615279

[18] Kroll, A., Monczak, K., Sorsche, D. & Rau, S. A luminescent ruthenium azide complex as a substrate for copper-catalyzed click reactions. *Eur. J. Inorg. Chem.* **2014**, 3462–3466 (2014).

[19] Huang, H. et al. Targeting nucleus DNA with a Cyclometalated Dipyridophenazineruthenium(II) Complex. *J. Med. Chem.* **57**, 8971–8983 (2014). 25313823 10.1021/jm501095r

[20] Cerfontaine, S. et al. MLCT Excited-State Behavior of Trinuclear Ruthenium(II) 2,2′-Bipyridine Complexes. *Inorg. Chem.* **60**, 366–379 (2021). 33351615 10.1021/acs.inorgchem.0c03004

[21] Sheeran, P. S., Luois, S., Dayton, P. A. & Matsunaga, T. O. Formulation and acoustic studies of a new phase-shift agent for diagnostic and therapeutic ultrasound. *Langmuir* **27**, 10412–10420 (2011). 21744860 10.1021/la2013705 PMC3164903

[22] Fa, N. et al. Effect of the antibiotic azithromycin on thermotropic behavior of DOPC or DPPC bilayers. *Chem. Phys. Lipids* **144**, 108–116 (2006). 17007828 10.1016/j.chemphyslip.2006.08.002

[23] Forier, K. et al. Probing the size limit for nanomedicine penetration into Burkholderia multivorans and Pseudomonas aeruginosa biofilms. *J. Control. Release* **195**, 21–28 (2014). 25125326 10.1016/j.jconrel.2014.07.061

[24] Sheeran, P. S., Luois, S. H., Mullin, L. B., Matsunaga, T. O. & Dayton, P. A. Design of ultrasonically-activatable nanoparticles using low boiling point perfluorocarbons. *Biomaterials* **33**, 3262–3269 (2012). 22289265 10.1016/j.biomaterials.2012.01.021 PMC3291020

[25] Wu, Q. et al. Investigation of the acoustic vaporization threshold of lipid-coated perfluorobutane nanodroplets using both high-speed optical imaging and acoustic methods. *Ultrasound Med. Biol.* **47**, 1826–1843 (2021). 33820668 10.1016/j.ultrasmedbio.2021.02.019

[26] Radhakrishnan, K., Holland, C. K. & Haworth, K. J. Scavenging dissolved oxygen via acoustic droplet vaporization. *Ultrason. Sonochem.* **31**, 394–403 (2016). 26964964 10.1016/j.ultsonch.2016.01.019 PMC4788814

[27] Young, B. C. et al. Antimicrobial resistance determinants are associated with Staphylococcus aureus bacteraemia and adaptation to the healthcare environment: a bacterial genome-wide association study. *Microb. Genom.* **7**, 10.1099/mgen.0.000700 (2021). 10.1099/mgen.0.000700 PMC8743558 34812717

[28] Young, B. C. et al. Microbial persistence, replacement and local antimicrobial therapy in recurrent bone and joint infection. *Antibiotics* **12**, 10.3390/antibiotics12040708 (2023). 10.3390/antibiotics12040708 PMC10135193 37107070

[29] Ipe, D. S. & Ulett, G. C. Evaluation of the in vitro growth of urinary tract infection-causing gram-negative and gram-positive bacteria in a proposed synthetic human urine (SHU) medium. *J. Microbiol. Methods* **127**, 164–171 (2016). 27312379 10.1016/j.mimet.2016.06.013

[30] Gomes, C., Ruiz-Roldán, L., Mateu, J., Ochoa, T. J. & Ruiz, J. Azithromycin resistance levels and mechanisms in Escherichia coli. *Sci. Rep.* **9**, 6089 (2019). 30988366 10.1038/s41598-019-42423-3 PMC6465286

[31] Kronvall, G. Normalized resistance interpretation as a tool for establishing epidemiological MIC susceptibility breakpoints. *J. Clin. Microbiol.* **48**, 4445–4452 (2010). 20926714 10.1128/JCM.01101-10 PMC3008453

[32] Haas, W., Pillar, C. M., Hesje, C. K., Sanfilippo, C. M. & Morris, T. W. Bactericidal activity of besifloxacin against staphylococci, Streptococcus pneumoniae and Haemophilus influenzae. *J. Antimicrob. Chemother.* **65**, 1441–1447 (2010). 20435780 10.1093/jac/dkq127 PMC2882870

[33] Cambau, E. et al. Target specificity of the new fluoroquinolone besifloxacin in Streptococcus pneumoniae, Staphylococcus aureus and Escherichia coli. *J. Antimicrob. Chemother.* **63**, 443–450 (2009). 19147516 10.1093/jac/dkn528

[34] Chew Ka, L., La, M.-V., Lin Raymond, T. P. & Teo Jeanette, W. P. Colistin and Polymyxin B Susceptibility Testing for Carbapenem-Resistant and mcr-Positive Enterobacteriaceae: Comparison of Sensititre, MicroScan, Vitek 2, and Etest with Broth Microdilution. *J. Clin. Microbiol* **55**, 2609–2616 (2017). 28592552 10.1128/JCM.00268-17 PMC5648698

[35] Yoshida, T. & Hiramatsu, K. Potent in vitro bactericidal activity of polymyxin B against methicillin-resistant Staphylococcus aureus (MRSA). *Microbiol Immunol.* **37**, 853–859 (1993). 8295564 10.1111/j.1348-0421.1993.tb01716.x

[36] Helfield, B., Chen, X., Watkins, S. C. & Villanueva, F. S. Biophysical insight into mechanisms of sonoporation. *Proc. Natl. Acad. Sci. USA* **113**, 9983–9988 (2016). 27551081 10.1073/pnas.1606915113 PMC5018802

[37] Kumaraswamy, M. et al. Standard susceptibility testing overlooks potent azithromycin activity and cationic peptide synergy against MDR Stenotrophomonas maltophilia. *J. Antimicrob. Chemother.* **71**, 1264–1269 (2016). 26832758 10.1093/jac/dkv487 PMC4830416

[38] Trimble, M. J., Mlynárčik, P., Kolář, M. & Hancock, R. E. Polymyxin: alternative mechanisms of action and resistance. *Cold Spring Harb Perspect. Med.* **6**, 10.1101/cshperspect.a025288 (2016). 10.1101/cshperspect.a025288 PMC5046685 27503996

[39] Hale, S. et al. Polymyxin B and ethylenediaminetetraacetic acid act synergistically against Pseudomonas aeruginosa and Staphylococcus aureus. *Microbiol. Spectr.* **12**, e01709–e01723 (2024). 38168683 10.1128/spectrum.01709-23 PMC10845947

[40] Whitfield, G. B., Marmont, L. S. & Howell, P. L. Enzymatic modifications of exopolysaccharides enhance bacterial persistence. *Front. Microbiol.* **6**, 471 (2015). 26029200 10.3389/fmicb.2015.00471 PMC4432689

[41] Li, L., Mendis, N., Trigui, H., Oliver, J. D. & Faucher, S. P. The importance of the viable but non-culturable state in human bacterial pathogens. *Front. Microbiol.* **5**, 258 (2014). 24917854 10.3389/fmicb.2014.00258 PMC4040921

[42] Pettit, R. K. et al. Microplate Alamar blue assay for Staphylococcus epidermidis biofilm susceptibility testing. *Antimicrob. Agents Chemother.* **49**, 2612–2617 (2005). 15980327 10.1128/AAC.49.7.2612-2617.2005 PMC1168683

[43] Bharatula, L. D., Marsili, E., Rice, S. A. & Kwan, J. J. Influence of high intensity focused ultrasound on the microstructure and c-di-GMP signaling of Pseudomonas aeruginosa biofilms. *Front. Microbiol.* **11**, 10.3389/fmicb.2020.599407 (2020). 10.3389/fmicb.2020.599407 PMC7769819 33384674

[44] Bau, L., Wu, Q., Ovenden, N. & Stride, E. P. Predicting the spontaneous vaporisation of superheated perfluorocarbondroplets. *J. Acoust. Soc. Am.* **155**, A249–A249 (2024).

[45] Macia, M. D., Rojo-Molinero, E. & Oliver, A. Antimicrobial susceptibility testing in biofilm-growing bacteria. *Clin. Microbiol. Infect.* **20**, 981–990 (2014). 24766583 10.1111/1469-0691.12651

[46] Toté, K. et al. Inhibitory efficacy of various antibiotics on matrix and viable mass of Staphylococcus aureus and Pseudomonas aeruginosa biofilms. *Int. J. Antimicrob. agents* **33**, 525–531 (2009). 19179053 10.1016/j.ijantimicag.2008.11.004

[47] Levison, M. E. & Levison, J. H. Pharmacokinetics and pharmacodynamics of antibacterial agents. *Infect. Dis. Clin. North Am.* **23**, 791–815 (2009). vii. 19909885 10.1016/j.idc.2009.06.008 PMC3675903

[48] Skogman, M. E., Vuorela, P. M. & Fallarero, A. Combining biofilm matrix measurements with biomass and viability assays in susceptibility assessments of antimicrobials against Staphylococcus aureus biofilms. *J. Antibiot.* **65**, 453–459 (2012). 10.1038/ja.2012.49 22739537

[49] Parsons, J. B. et al. In-patient evolution of a high-persister Escherichia coli strain with reduced in vivo antibiotic susceptibility. *Proc. Natl. Acad. Sci. USA* **121**, e2314514121 (2024). 38190524 10.1073/pnas.2314514121 PMC10801923

[50] Shultis, M. W., Mulholland, C. V. & Berney, M. Are all antibiotic persisters created equal? *Front. Cell. Infect. Microbiol.* **12**, 933458 (2022). 36061872 10.3389/fcimb.2022.933458 PMC9428696

[51] Song, S. & Wood, T. K. Are we really studying persister cells? *Environ. Microbiol. Rep.* **13**, 3–7 (2021). 32363793 10.1111/1758-2229.12849

[52] Yang, S. et al. Antibiotic regimen based on population analysis of residing persister cells eradicates Staphylococcus epidermidis biofilms. *Sci. Rep.* **5**, 18578 (2015). 26687035 10.1038/srep18578 PMC4685274

[53] Cañas-Duarte, S. J., Restrepo, S. & Pedraza, J. M. Novel protocol for persister cells isolation. *PLoS ONE* **9**, e88660 (2014). 24586365 10.1371/journal.pone.0088660 PMC3931647

[54] Balaban, N. Q., Merrin, J., Chait, R., Kowalik, L. & Leibler, S. Bacterial persistence as a phenotypic switch. *Science* **305**, 1622–1625 (2004). 15308767 10.1126/science.1099390

[55] Ayrapetyan, M., Williams, T. C., Baxter, R. & Oliver, J. D. Viable but nonculturable and persister cells coexist stochastically and are induced by human serum. *Infect. Immun.* **83**, 4194–4203 (2015). 26283335 10.1128/IAI.00404-15 PMC4598401

[56] Chen, H. et al. The progress of type II persisters of Escherichia coli O157:H7 to a non-culturable state during prolonged exposure to antibiotic stress with revival being aided through acid-shock treatment and provision of methyl pyruvate. *Can. J. Microbiol.* **67**, 518–528 (2020). 33125853 10.1139/cjm-2020-0339

[57] Orman, M. A., Henry, T. C., DeCoste, C. J. & Brynildsen, M. P. Analyzing persister physiology with fluorescence-activated cell sorting. *Methods Mol. Biol.* **1333**, 83–100 (2016). 26468102 10.1007/978-1-4939-2854-5_8 PMC4908830

[58] Kerstens, M. et al. A flow cytometric approach to quantify biofilms. *Folia Microbiol.* **60**, 335–342 (2015). 25948317 10.1007/s12223-015-0400-4

[59] Monsen, T., Lövgren, E., Widerström, M. & Wallinder, L. In vitro effect of ultrasound on bacteria and suggested protocol for sonication and diagnosis of prosthetic infections. *J. Clin. Microbiol.* **47**, 2496–2501 (2009). 19535525 10.1128/JCM.02316-08 PMC2725697

[60] Fleming, D. & Rumbaugh, K. The consequences of biofilm dispersal on the host. *Sci. Rep.* **8**, 10.1038/s41598-018-29121-2 (2018). 10.1038/s41598-018-29121-2 PMC6048044 30013112

[61] Barraud, N., Kjelleberg, S. & Rice Scott, A. Dispersal from microbial biofilms. *Microbiol. Spectr.* **3**, 10.1128/microbiolspec.mb-0015-2014 (2015). 10.1128/microbiolspec.MB-0015-2014 27337281

[62] Woo, J. K. K., Webb, J. S., Kirov, S. M., Kjelleberg, S. & Rice, S. A. Biofilm dispersal cells of a cystic fibrosis Pseudomonas aeruginosa isolate exhibit variability in functional traits likely to contribute to persistent infection. *FEMS Immunol. Med. Microbiol.* **66**, 251–264 (2012). 22765766 10.1111/j.1574-695X.2012.01006.x

[63] Miao, L., Liu, W., Qiao, Q., Li, X. & Xu, Z. Fluorescent antibiotics for real-time tracking of pathogenic bacteria. *J. Pharm. Anal.* **10**, 444–451 (2020). 33133728 10.1016/j.jpha.2020.09.003 PMC7591806

[64] Prochnow, H. et al. Subcellular quantification of uptake in gram-negative bacteria. *Anal. Chem.* **91**, 1863–1872 (2019). 30485749 10.1021/acs.analchem.8b03586

[65] Čurman, D. et al. Spectral properties of Eu(III) compound with antibacterial agent ciprofloxacin (cfqH). Crystal structure of [Eu(cfqH)(cfq)(H2O)4]Cl2·4.55H2O. *Polyhedron* **27**, 1489–1496 (2008).

[66] Uivarosi, V. Metal complexes of quinolone antibiotics and their applications: an update. *Molecules* **18**, 11153–11197 (2013). 24029748 10.3390/molecules180911153 PMC6269848

[67] Antibiotic-metal complexes in the detection of gram-positive bacteria andother biological analytes. https://patents.google.com/patent/US20050026813A1/en (2003).

[68] Polishchuk, A. V., Karaseva, E. T., Medkov, M. A. & Karasev, V. E. Europium(III) compounds with cyprofloxacine and norfloxacine: spectral and luminescent properties and antibacterial activity. *Russ. J. Coord. Chem.* **30**, 828–831 (2004).

[69] Olstein, A. (D. M. H., MN, US), Feirtag, J. (St. Paul, MN, US). Antibiotic-metal complexes in the detection of gram-positive bacteria and other biological analytes. United States patent (2005).

[70] Zheng, X. et al. The cell envelope of Staphylococcus aureus selectively controls the sorting of virulence factors. *Nat. Commun.* **12**, 6193 (2021). 34702812 10.1038/s41467-021-26517-z PMC8548510

[71] Kosztołowicz, T. & Metzler, R. Diffusion of antibiotics through a biofilm in the presence of diffusion and absorption barriers. *Phys. Rev. E* **102**(3-1), 032408 (2020). 33075880 10.1103/PhysRevE.102.032408

[72] Hancock, R. E. W. & Bell, A. Antibiotic uptake into gram-negative bacteria. *Eur. J. Clin. Microbiol. Infect. Dis.* **7**, 713–720 (1988). 2850910 10.1007/BF01975036

[73] Cama, J. et al. Single-cell microfluidics facilitates the rapid quantification of antibiotic accumulation in Gram-negative bacteria. *Lab Chip* **20**, 2765–2775 (2020). 32613221 10.1039/d0lc00242a PMC7953842

[74] Pandeya, A., Alegun, O., Cai, Y. & Wei, Y. Distribution of fluoroquinolones in the two aqueous compartments of Escherichia coli. *Biochem. Biophys. Rep.* **24**, 100849 (2020). 33235925 10.1016/j.bbrep.2020.100849 PMC7670238

[75] Zhang, L., Dhillon, P., Yan, H., Farmer, S. & Hancock, R. E. Interactions of bacterial cationic peptide antibiotics with outer and cytoplasmic membranes of Pseudomonas aeruginosa. *Antimicrob. Agents Chemother.* **44**, 3317–3321 (2000). 11083634 10.1128/aac.44.12.3317-3321.2000 PMC90199

[76] Ciro, Y., Rojas, J., Oñate-Garzon, J. & Salamanca, C. H. Synthesis, characterisation and biological evaluation of ampicillin-chitosan-polyanion nanoparticles produced by ionic gelation and polyelectrolyte complexation assisted by high-intensity sonication. *Polymers* **11**, 10.3390/polym11111758 (2019). 10.3390/polym11111758 PMC6918291 31731554

[77] Ansari, M. A. et al. Interaction of Al(2)O(3) nanoparticles with Escherichia coli and their cell envelope biomolecules. *J. Appl. Microbiol.* **116**, 772–783 (2014). 24354999 10.1111/jam.12423

[78] Jiang, Y. et al. Hydrophilic nanoparticles that kill bacteria while sparing mammalian cells reveal the antibiotic role of nanostructures. *Nat. Commun.* **13**, 197 (2022). 35017467 10.1038/s41467-021-27193-9 PMC8752835

[79] Lonhienne, T. G. A. et al. Endocytosis-like protein uptake in the bacterium Gemmata obscuriglobus. *Proc. Natl. Acad. Sci. USA* **107**, 12883–12888 (2010). 20566852 10.1073/pnas.1001085107 PMC2919973

[80] Rapoport, N., Smirnov, A. I., Timoshin, A., Pratt, A. M. & Pitt, W. G. Factors affecting the permeability of Pseudomonas aeruginosa cell walls toward lipophilic compounds: effects of ultrasound and cell age. *Arch. Biochem. Biophys.* **344**, 114–124 (1997). 9244388 10.1006/abbi.1997.0176

[81] Sirsi, S. R. & Borden, M. A. State-of-the-art materials for ultrasound-triggered drug delivery. *Adv. Drug Deliv. Rev.* **72**, 3–14 (2014). 24389162 10.1016/j.addr.2013.12.010 PMC4041842

[82] Tang, T. et al. The drug tolerant persisters of Riemerella anatipestifer can be eradicated by a combination of two or three antibiotics. *BMC Microbiol* **18**, 137 (2018). 30340538 10.1186/s12866-018-1303-8 PMC6194556

[83] Murray, C. J. L. et al. Global burden of bacterial antimicrobial resistance in 2019: a systematic analysis. *The Lancet*10.1016/S0140-6736(21)02724-0 (2022). 10.1016/S0140-6736(21)02724-0 PMC8841637 35065702

[84] Sartor, V., Irvoas, J., Bordeau, G. & Chouini-Lalanne, N. Multivalent Azide-Functionalized Polypyridyl Ruthenium Complexes and Their DNA Conjugates through Click Chemistry. *Eur. J. Inorg. Chem.* **2017**, 2661–2670 (2017).

[85] Sultana, N. Synthesis characterization and antimicrobial activities of azithromycin metal complexes. *Mod. Chem. Appl.* **02**, 10.4172/2329-6798.1000133 (2014).

[86] Djokic, S., Vajtner, Z., Krnjevic, H., Lopotar, N. & Kolacny-Babic, L. Complexes and chelates of azithromycin with bivalent and/or trivalent metals and their use as antiulcer. US patent US 5498699 A (1996).

[87] Olstein Alan, D. & Feirtag Joellen, M. Antibiotic-metal complexes in the detection of gram-negative bacteria and other biological analytes. WO patent WO 2001/027628 A1 (2001).

[88] Bonifait, L., Grignon, L. & Grenier, D. Fibrinogen induces biofilm formation by Streptococcus suis and enhances its antibiotic resistance. *Appl. Environ. Microbiol.* **74**, 4969–4972 (2008). 18539785 10.1128/AEM.00558-08 PMC2519328

[89] Stamm, J. et al. Development of an artificial synovial fluid useful for studying Staphylococcus epidermidis joint infections. *Front. Cell. Infect. Microbiol.* **12**, 948151 (2022). 35967857 10.3389/fcimb.2022.948151 PMC9374174

[90] Au-Kirchner, S. et al. Use of artificial sputum medium to test antibiotic efficacy against pseudomonas aeruginosa in conditions more relevant to the cystic fibrosis lung. *J.Vis. Exp.* e3857. 10.3791/3857 (2012). 10.3791/3857 PMC3471314 22711026

[91] Lutheryn, G. et al. Bactericidal and anti-biofilm effects of uncharged and cationic ultrasound-responsive nitric oxide microbubbles on Pseudomonas aeruginosa biofilms. *Front. Cell. Infect. Microbiol.* **12**, 956808 (2022). 35992170 10.3389/fcimb.2022.956808 PMC9386126

[92] Dankert, J., Holloway, Y., Joldersma, W. & Hess, J. Importance of minimizing carry-over effect at subculture in the detection of penicillin-tolerant viridans group streptococci. *Antimicrob. Agents Chemother.* **23**, 614–616 (1983). 6602588 10.1128/aac.23.4.614 PMC184711

[93] Hacek, D. M., Dressel, D. C. & Peterson, L. R. Highly reproducible bactericidal activity test results by using a modified National Committee for Clinical Laboratory Standards broth macrodilution technique. *J. Clin. Microbiol.* **37**, 1881–1884 (1999). 10325341 10.1128/jcm.37.6.1881-1884.1999 PMC84976

[94] Eagle, H. & Musselman, A. D. The rate of bactericidal action of penicillin in vitro as a function of its concentration, and its paradoxically reduced activity at high concentrations against certain organisms. *J. Exp. Med.* **88**, 99–131 (1948). 18871882 10.1084/jem.88.1.99 PMC2135799

[95] Stewart, P. S. & Parker, A. E. Measuring antimicrobial efficacy against biofilms: a meta-analysis. *Antimicrob. Agents Chemother.* **63**, 10.1128/aac.00020-19 (2019). 10.1128/AAC.00020-19 PMC6496104 30803974

[96] Kim, J. S., Yamasaki, R., Song, S., Zhang, W. & Wood, T. K. Single cell observations show persister cells wake based on ribosome content. *Environ. Microbiol.* **20**, 2085–2098 (2018). 29528544 10.1111/1462-2920.14093

