# Supplementary figures and images for: Repurposing antimicrobials with ultrasound-triggered nanoscale systems for targeted biofilm drug delivery
Source: NPJ Antimicrob Resist. 2025 Apr 1;3:22. doi: 10.1038/s44259-025-00086-3 (PMC11962098; doi:10.1038/s44259-025-00086-3)

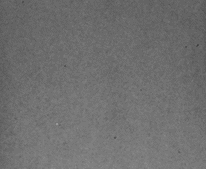

Supplement: Supplementary file 1 — supplementary video 1 [file 44259_2025_86_MOESM1_ESM.gif]

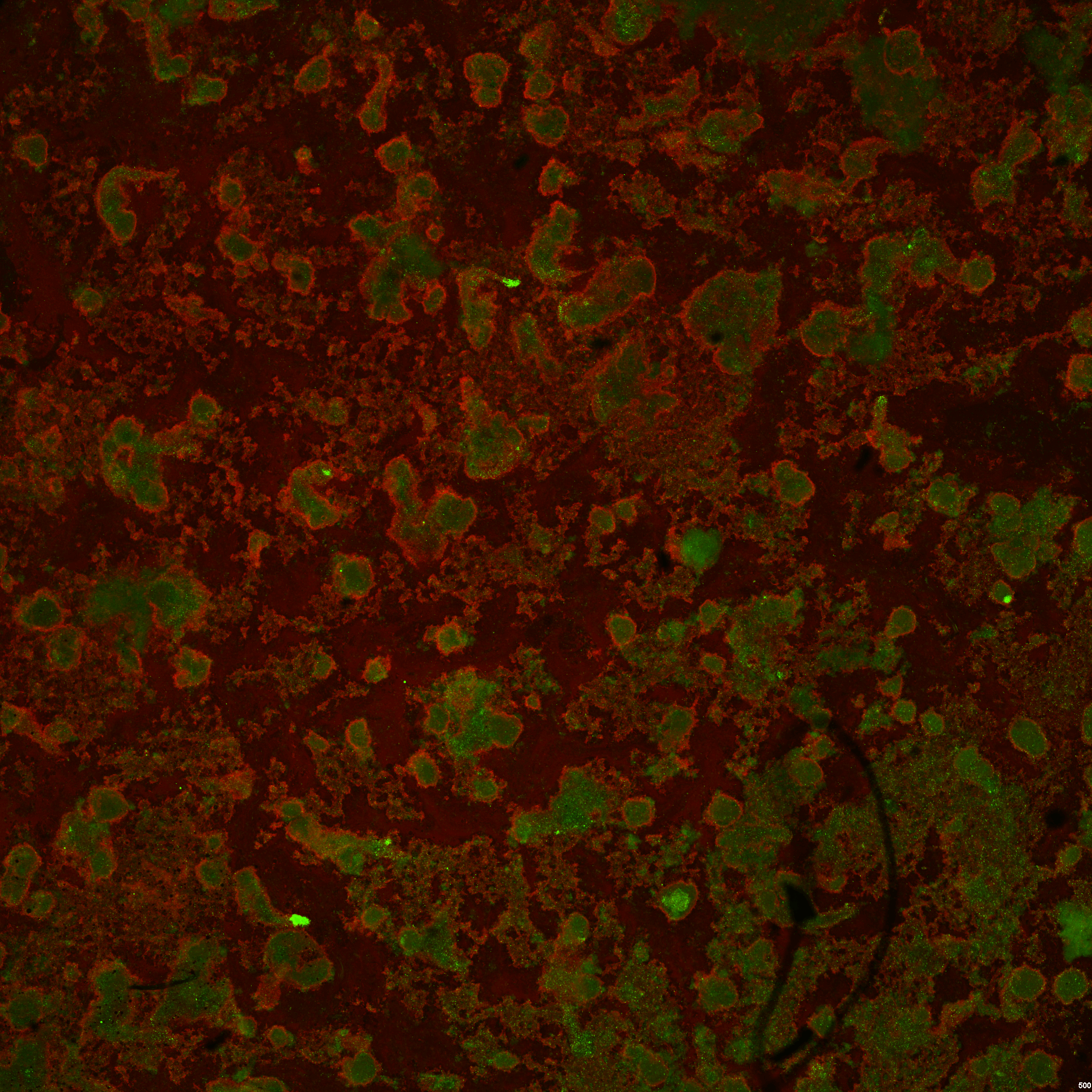

Supplement: Supplementary file 2 — supplementary video 2 [file 44259_2025_86_MOESM2_ESM.gif]
