# Supplementary material for: Repurposing antimicrobials with ultrasound-triggered nanoscale systems for targeted biofilm drug delivery
Source: NPJ Antimicrob Resist. 2025 Apr 1;3:22. doi: 10.1038/s44259-025-00086-3 (PMC11962098; doi:10.1038/s44259-025-00086-3)
Supplement: Supplementary file 3 — Supporting Information [file 44259_2025_86_MOESM3_ESM.pdf]

Supporting Information

Biofilm Drug Delivery: Repurposing Antimicrobials through Ultrasound-Triggered Nanoscale Systems Against Clinical Isolates

Victor Choi, Dario Carugo, and Eleanor Stride*

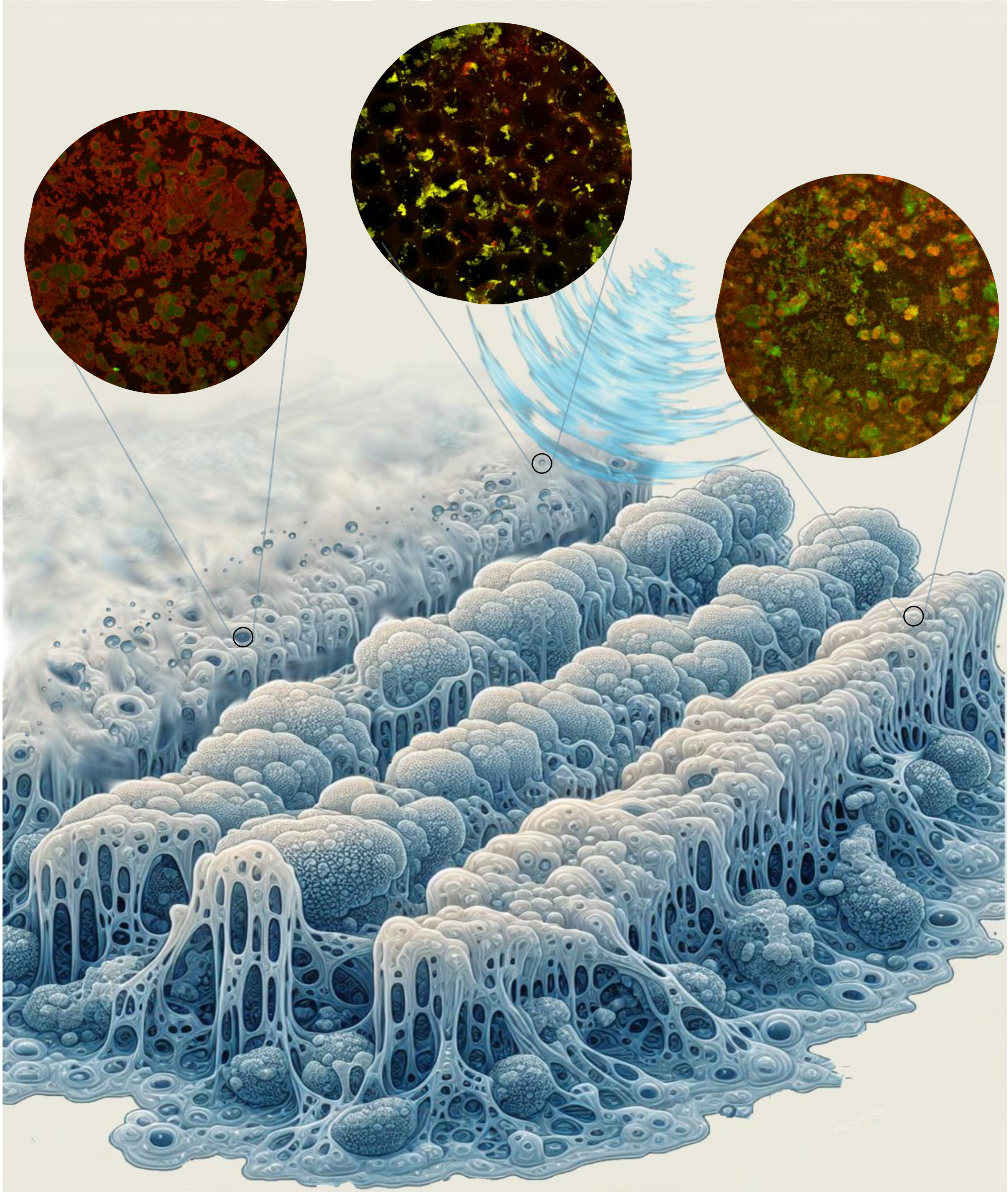

Figure S1: Schematic of nanodroplet-focused ultrasound on biofilm infections. Right-most inset displays a representative biofilm, untreated (green = cells, red = EPS). Middle inset displays a biofilm following treatment (green = cells, red = EPS, yellow = droplets). Left-most inset displays nanodroplets (red) accumulating within biofilm-embedded cells.

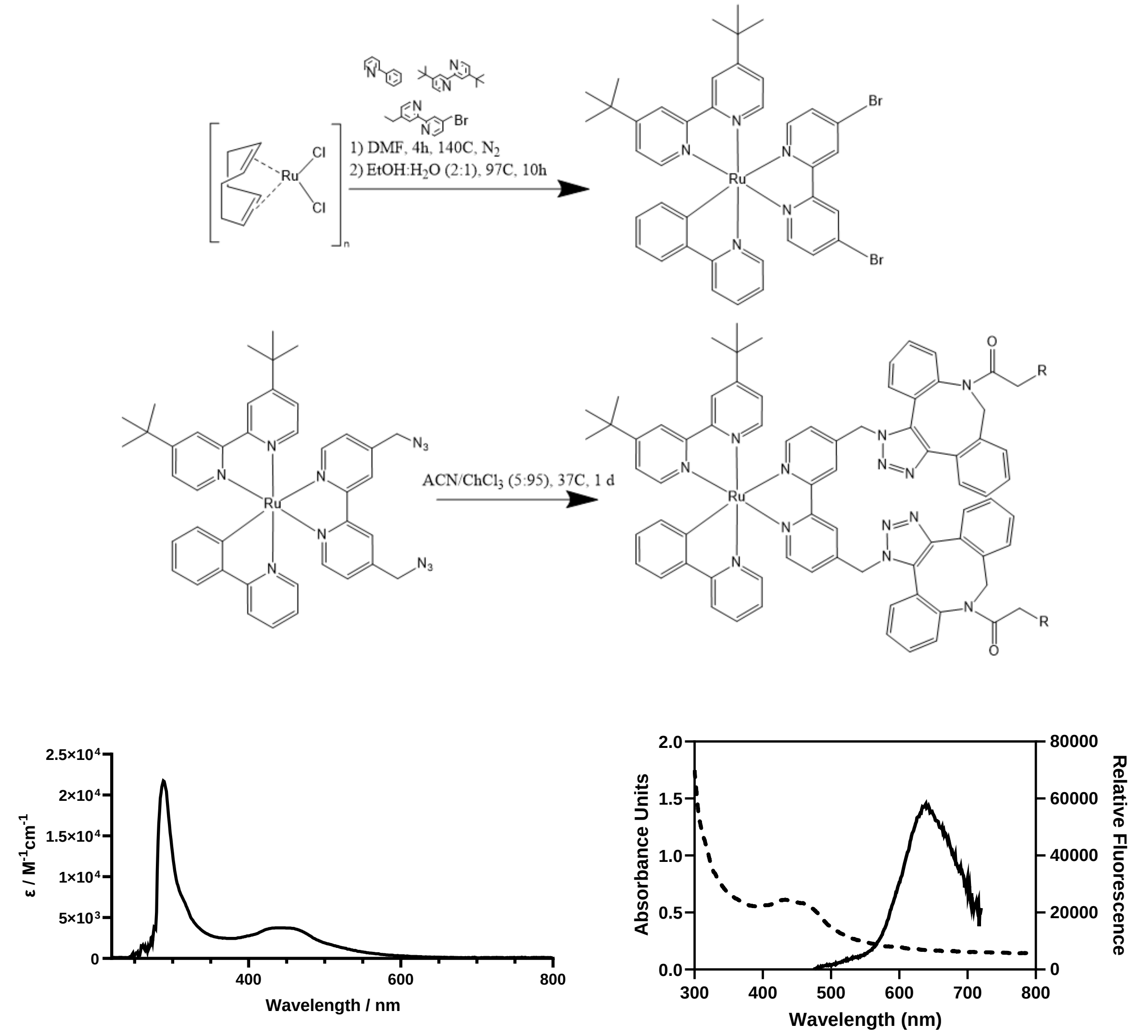

Figure S2: Synthesis of the ruthenium polypyridyl complex through modification of established protocols (top). Successful synthesis was confirmed using 1H NMR, 13C NMR, HPLC-ESI-MS (m/z [M]+: 792.27), and optical characterization. Complexes displayed the typical MLCT absorption at 456 nm (bottom-left), which when excited, emitted fluorescence at 624 nm (bottom-right).

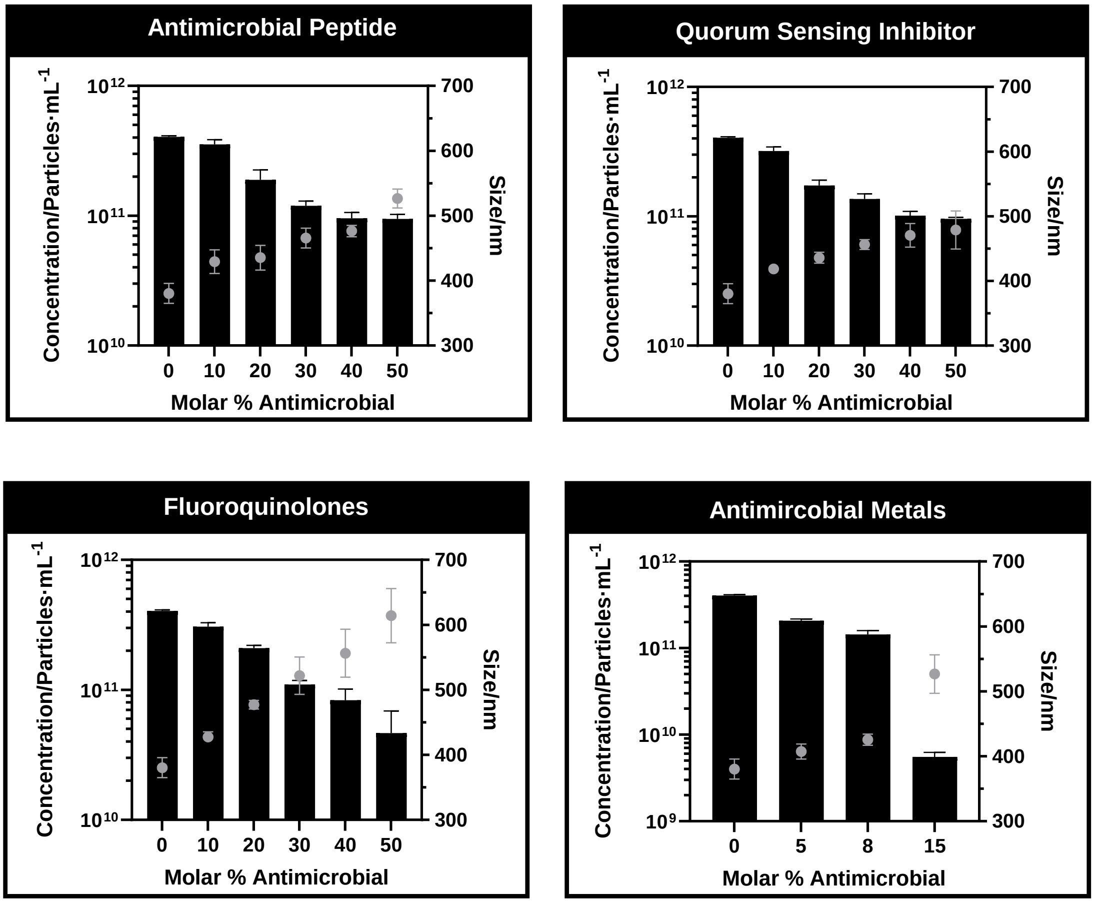

Figure S3: Size and concentration of precursor microbubbles loaded with antimicrobials, as measured through interferometry, dynamic light scattering, and electro-impedance volumetric sensing. Bars represent the concentration of microbubbles/mL whereas symbols represent the mean size. An average of five replicates was taken, with three technical replicates per technique.

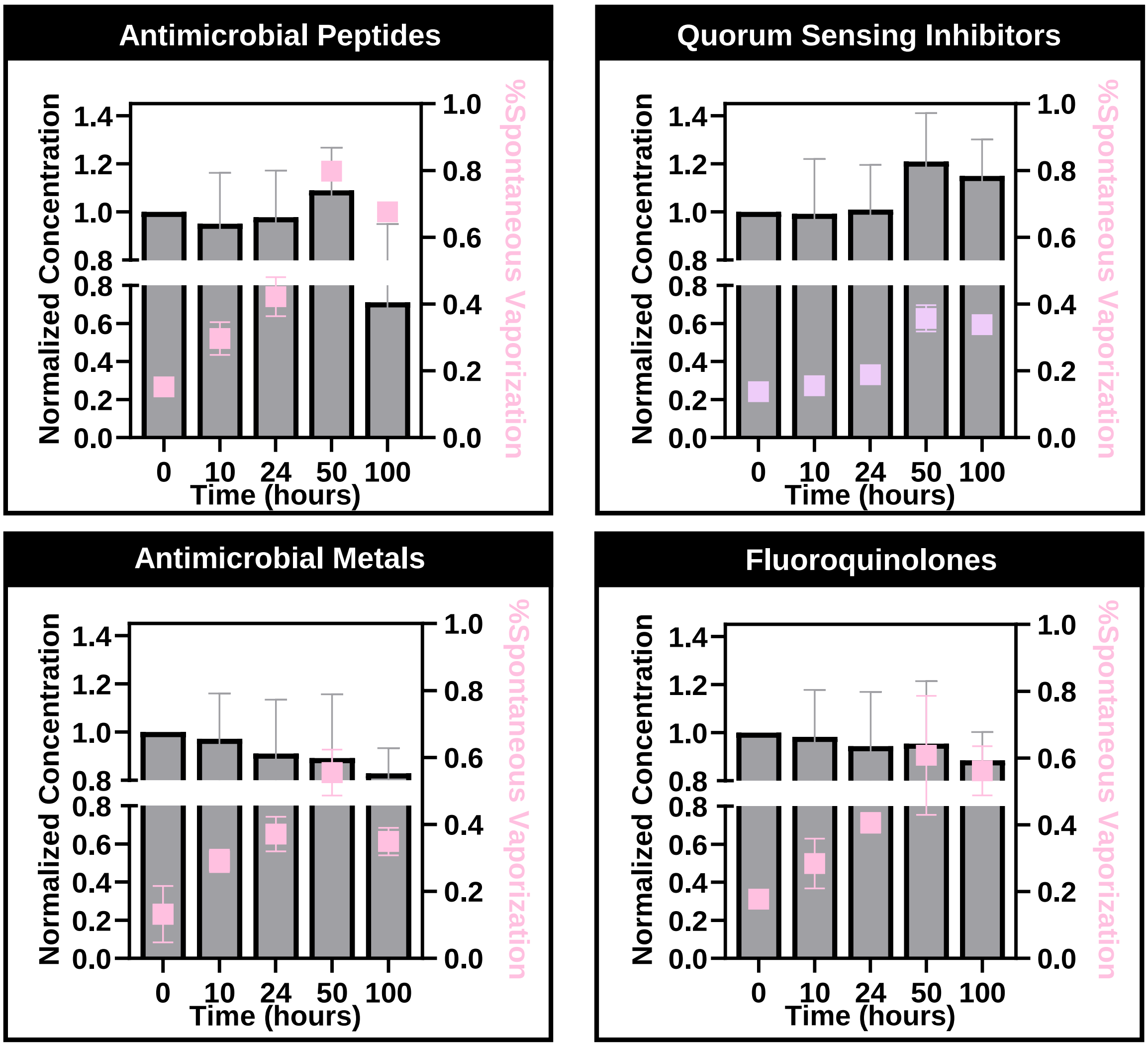

Figure S4: Serum stability of antimicrobial-loaded nanodroplets in 50% fetal bovine serum (FBS) and 50% PBS. Measurements were taken at the time points indicated on interferometry, dynamic light scattering, and electro-impedance volumetric zone sensing. Bars represent the normalized concentration to time 0 whereas symbols represent the mean diameter of the particle.

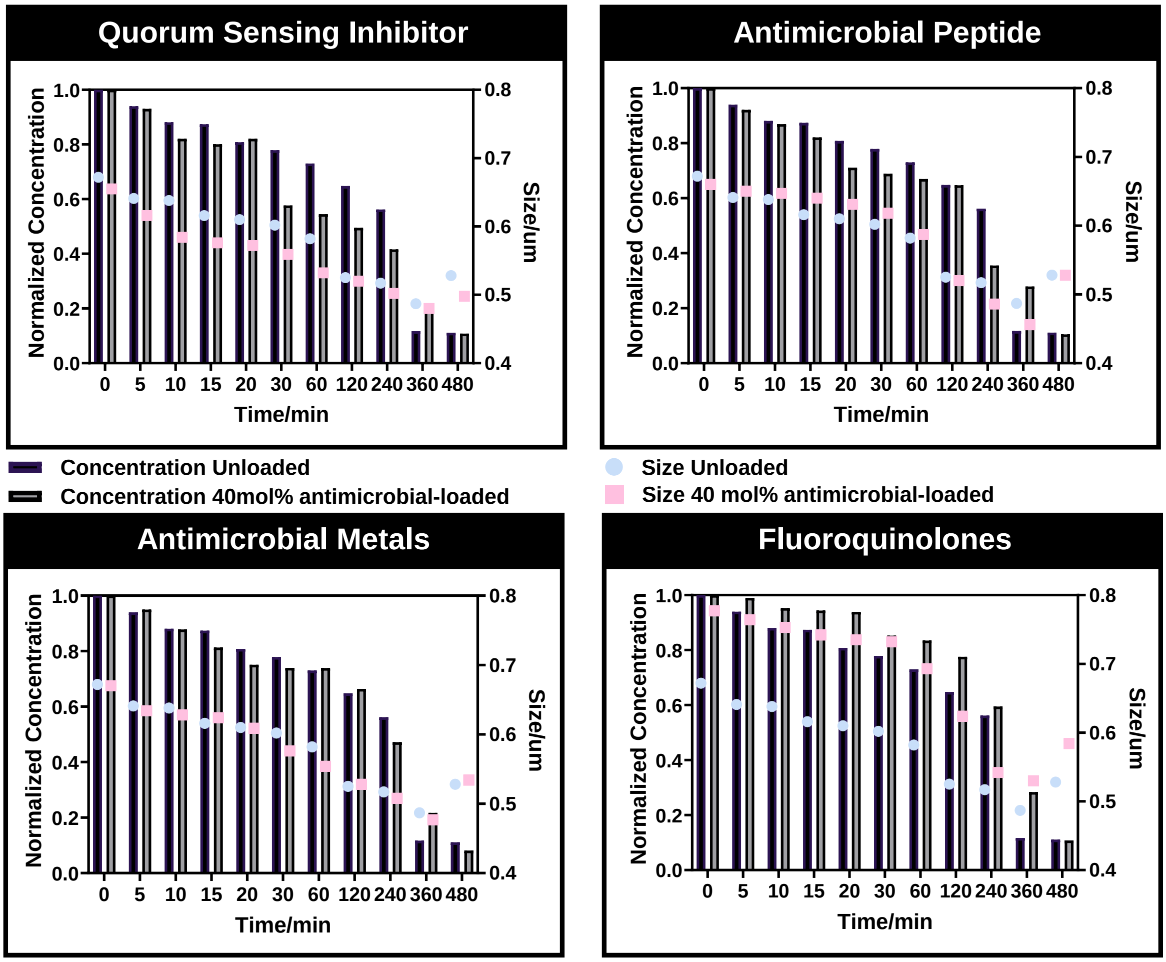

Figure S5: Stability of precursor microbubbles in 50% fetal bovine serum (FBS) and 50% PBS. Measurements were taken at the time points indicated on electro-impedance volumetric zone sensing only. Bars represent the normalized concentration to time 0 whereas symbols represent the mean diameter of the particle. Both unloaded and 40mol% antimicrobial loaded particles were measured across all time points.

	Phosphate 7.4	Carbonate 7.4	Acetate 7.4	Plasma 7.4
AzNDs	-29.03 ± 2.11	-30.63 ± 0.63	-5.17 ± 1.87	-2.20 ± 0.13
BfNDs	-26.57 ± 2.16	-14.43 ± 1.67	-4.61 ± 0.62	-2.48 ± 1.65
PbNDs	-1.69 ± 0.47	-3.89 ± 1.72	-1.44 ± 0.44	8.46 ± 4.11
RuNDs	-26.6 ± 2.55	-25.93 ± 1.96	-2.46 ± 1.45	-2.36 ± 2.16

Figure S6: Zeta potentials of antimicrobial-loaded nanodroplets in different physiologically relevant buffers at pH 7.4. Each buffer was diluted to 10 mM prior to measurement and each measurement was conducted in triplicate, with three technical replicates per run.

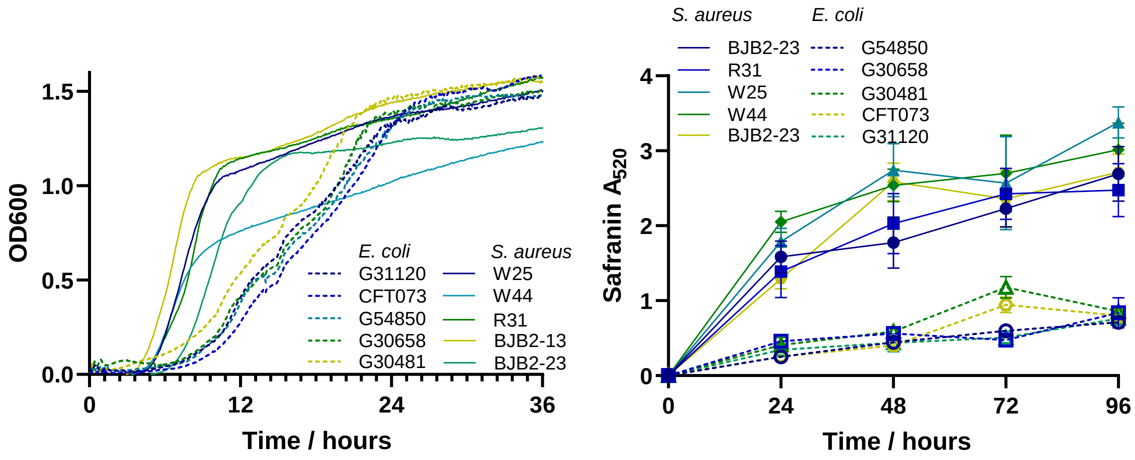

Figure S7: Planktonic growth kinetics of *E. coli* and *S. aureus* clinical isolates as grown on either urine or brain heart infusion, respectively (left). Measurements were done at 600 nm to measure the turbidity of each sample, representing bacterial growth. Biofilm growth kinetics of the same clinical isolates (right) were measured every 24 hours using safranin. Three biological replicates were conducted for each isolate at each time point, with three technical replicates performed per sample.

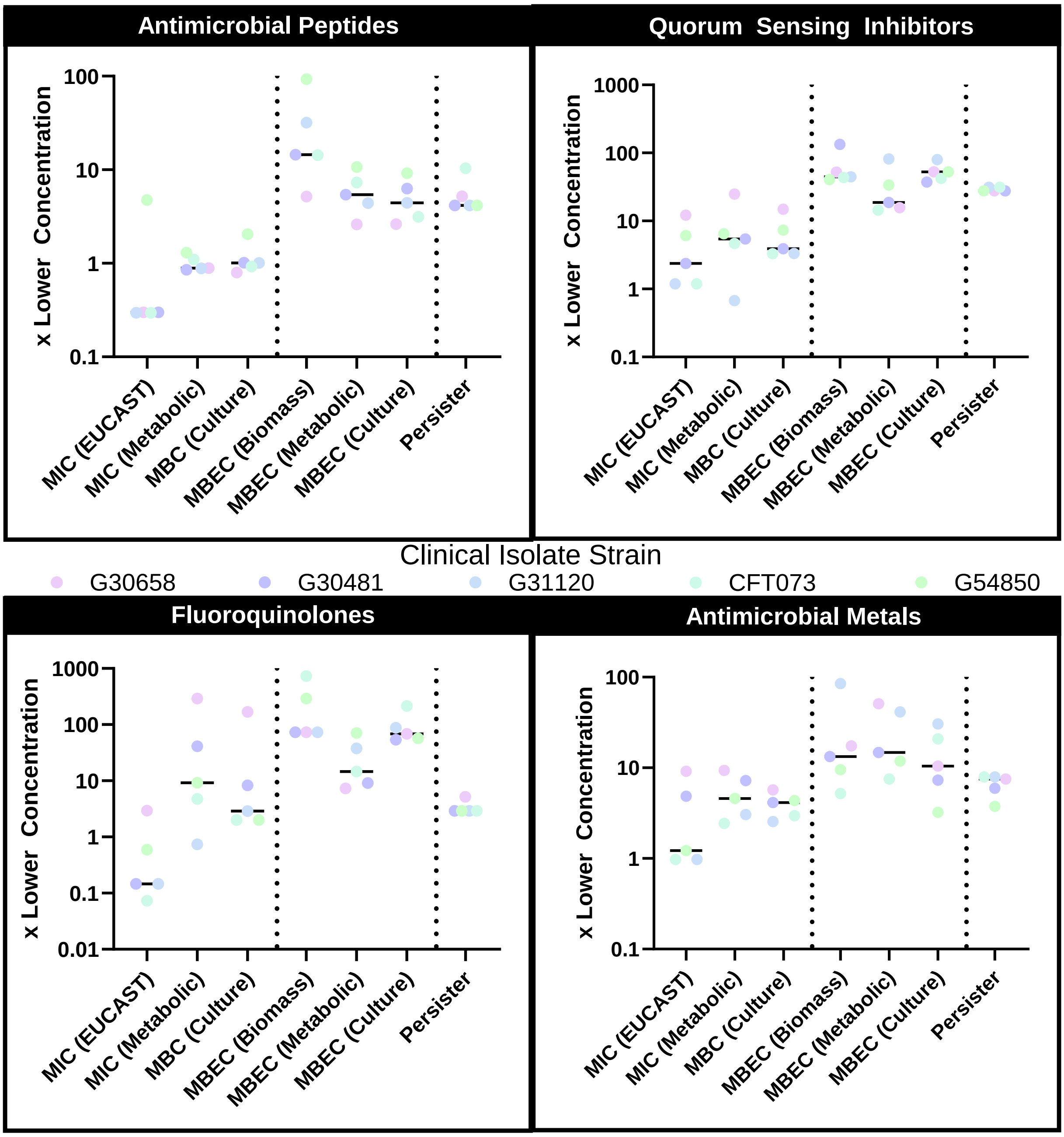

Figure S8: Efficacy comparison of antimicrobial-loaded ND/FUS in *E. coli* clinical isolates relative to free drug across various antimicrobial classes. Plots depict the fold reduction in antimicrobial concentration required by the nanodroplet platform to achieve the Minimum Inhibitory Concentration (MIC) per EUCAST standards and metabolic viability (resazurin), Minimum Bactericidal Concentration (MBC; defined as a 3-log reduction in CFU/mL), and Minimum Biofilm Eradication Concentration (MBEC). MBEC metrics include biomass reduction (90% reduction in safranin), metabolic viability (80% reduction in resazurin) and culturability (3-log reduction in CFU/mL). Persister assays are conducted and defined as the lowest concentration needed for complete eradication below the detection limit of 10^0 CFU/mL. Each colored symbol corresponds to a different uropathogenic *E. coli* clinical isolate strain, with each isolate represented by three biological replicates, each with three technical replicates per measurement technique. Horizontal black lines indicate median values. Dotted black lines differentiate between planktonic, biofilm, and persister cell conditions.

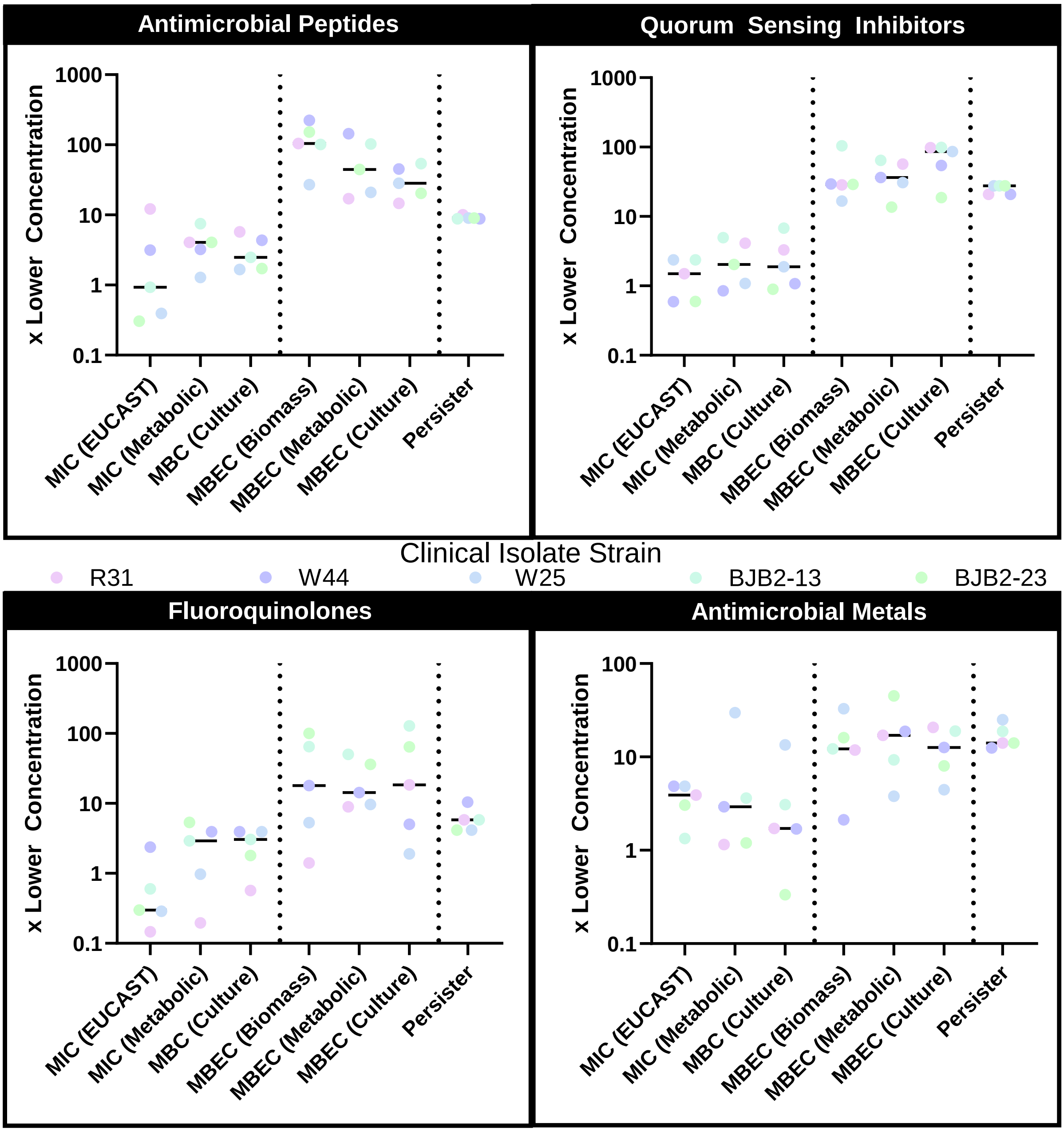

Figure S9: Efficacy comparison of antimicrobial-loaded ND/FUS in *S. aureus* clinical isolates relative to free drug across various antimicrobial classes. Plots depict the fold reduction in antimicrobial concentration required ND/FUS compared to free drug to achieve the Minimum Inhibitory Concentration (MIC) per EUCAST standards and metabolic viability (resazurin), Minimum Bactericidal Concentration (MBC; defined as a 3-log reduction in CFU/mL), and Minimum Biofilm Eradication Concentration (MBEC). MBEC metrics include biomass reduction (90% reduction in safranin), metabolic viability (80% reduction in resazurin) and culturability (3-log reduction in CFU/mL). Persister assays are conducted and defined as the lowest concentration needed for complete eradication below the detection limit of 10^0 CFU/mL. Each colored symbol corresponds to a different *S. aureus* clinical isolate strain, with each isolate represented by three biological replicates, each with three technical replicates per measurement technique. Horizontal black lines indicate median values. Dotted black lines differentiate between planktonic, biofilm, and persister cell conditions

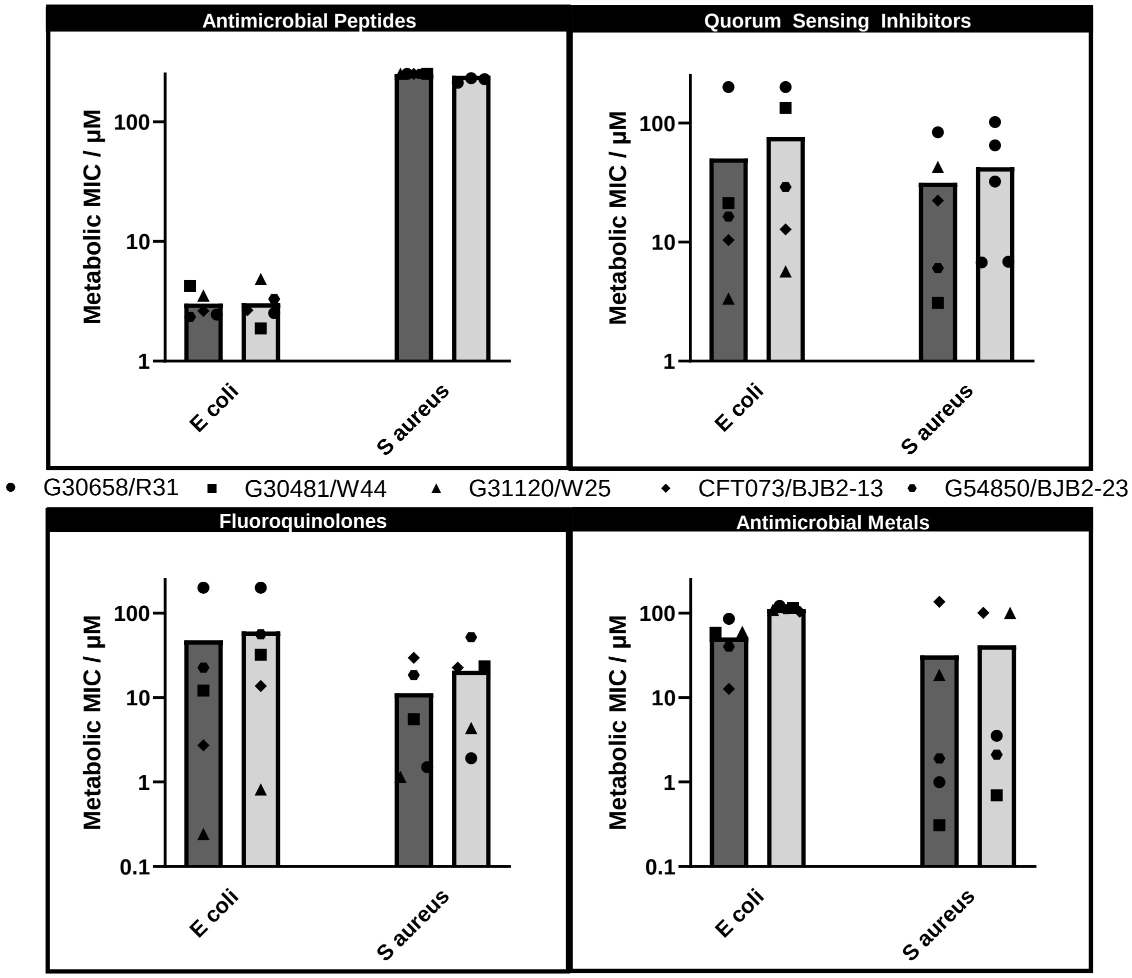

Figure S10: Metabolic minimum inhibitory concentration (80% reduction in resazurin) of antimicrobial lipid suspensions (dark grey) compared against antimicrobial-loaded nanodroplets (light grey). Each point represents a different clinical isolate, performed in triplicate. Due to concentration limitations, a maximum of 250 µM was assessed and can be observed as a plateau in certain drug/isolate combinations.

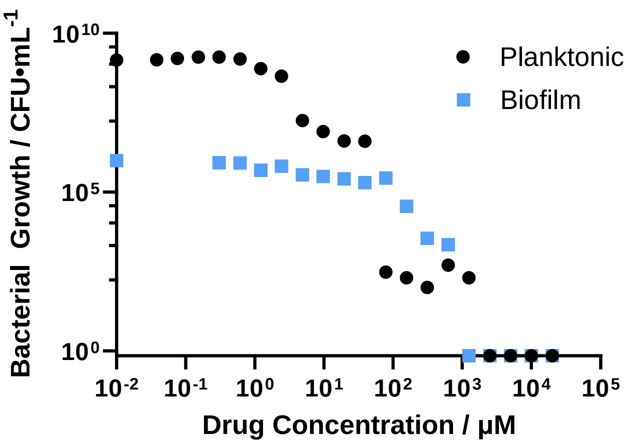

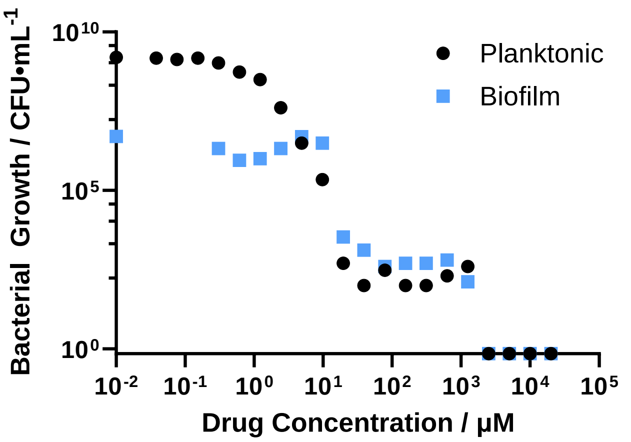

Figure S11: Representative biphasic/triphasic killing curve observed across samples with increasing antimicrobial concentration against *E. coli* (top) and *S. aureus* (bottom) clinical isolates. A minimum of two inflection points can be observed, correlating to the bactericidal concentration as well as the persister elimination concentration.

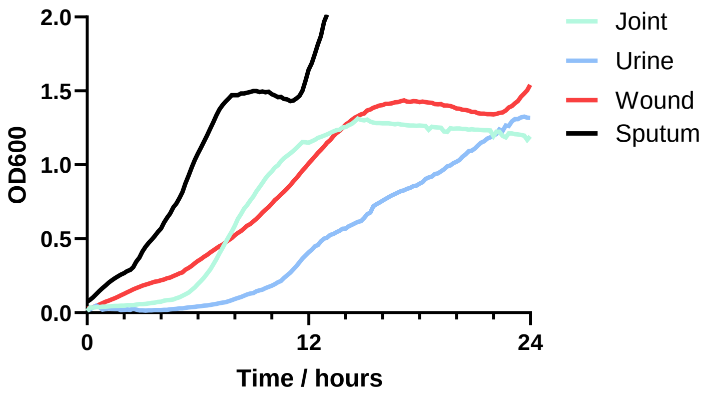

Figure S12: Planktonic growth kinetics of *E. coli*, *S. aureus*, and *P. aeruginosa* when grown in synthetic human urine, synovial fluid, artificial wound medium, or artificial sputum. Measurements were done at 600 nm to measure the turbidity of each sample, representing bacterial growth. To account for the natural turbidity of the artificial wound medium, measurements were taken at 700 nm instead.

	Absorbance
Eu-Azithromycin	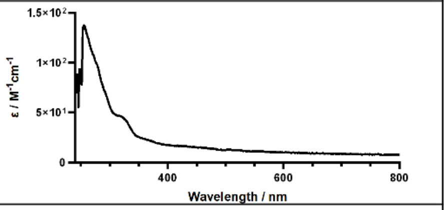
Eu-Besifloxacin	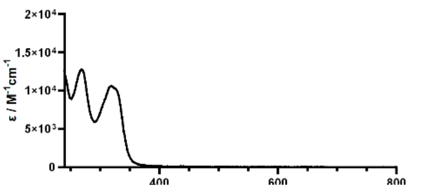
Eu-Polymyxin B	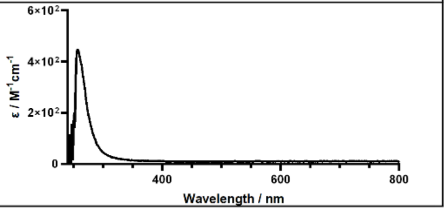

.

Figure S13: Optical characterization of europium-chelated antimicrobials.

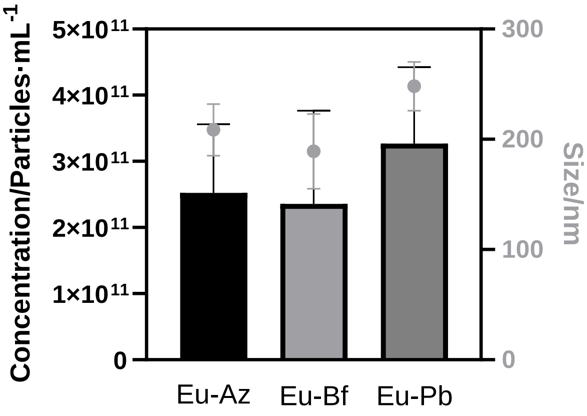

Figure S14: Concentration and size of europium-chelated antimicrobial-loaded nanodroplets. Concentrations are expressed as bars, as measured using interferometry, dynamic light scattering, and electro-impedance volumetric zone sensing. Sizes are expressed as symbols measured using the same. Each measurement was performed in triplicate.

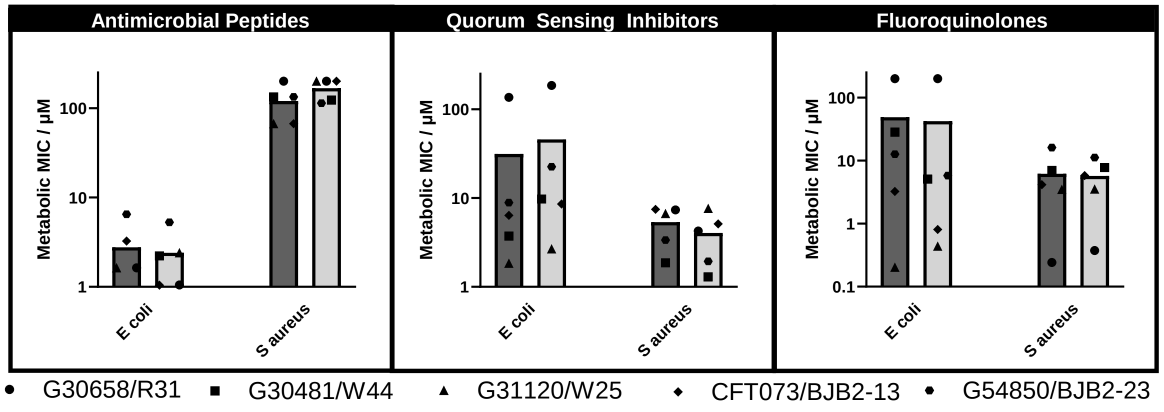

Figure S15: Metabolic minimum inhibitory concentration (80% reduction in resazurin) of free drug (dark grey) compared against europium-chelated antimicrobials (light grey). Each point represents a different clinical isolate, performed in triplicate. Due to concentration limitations, a maximum of 250 µM was assessed and can be observed as a plateau in certain drug/isolate combinations.

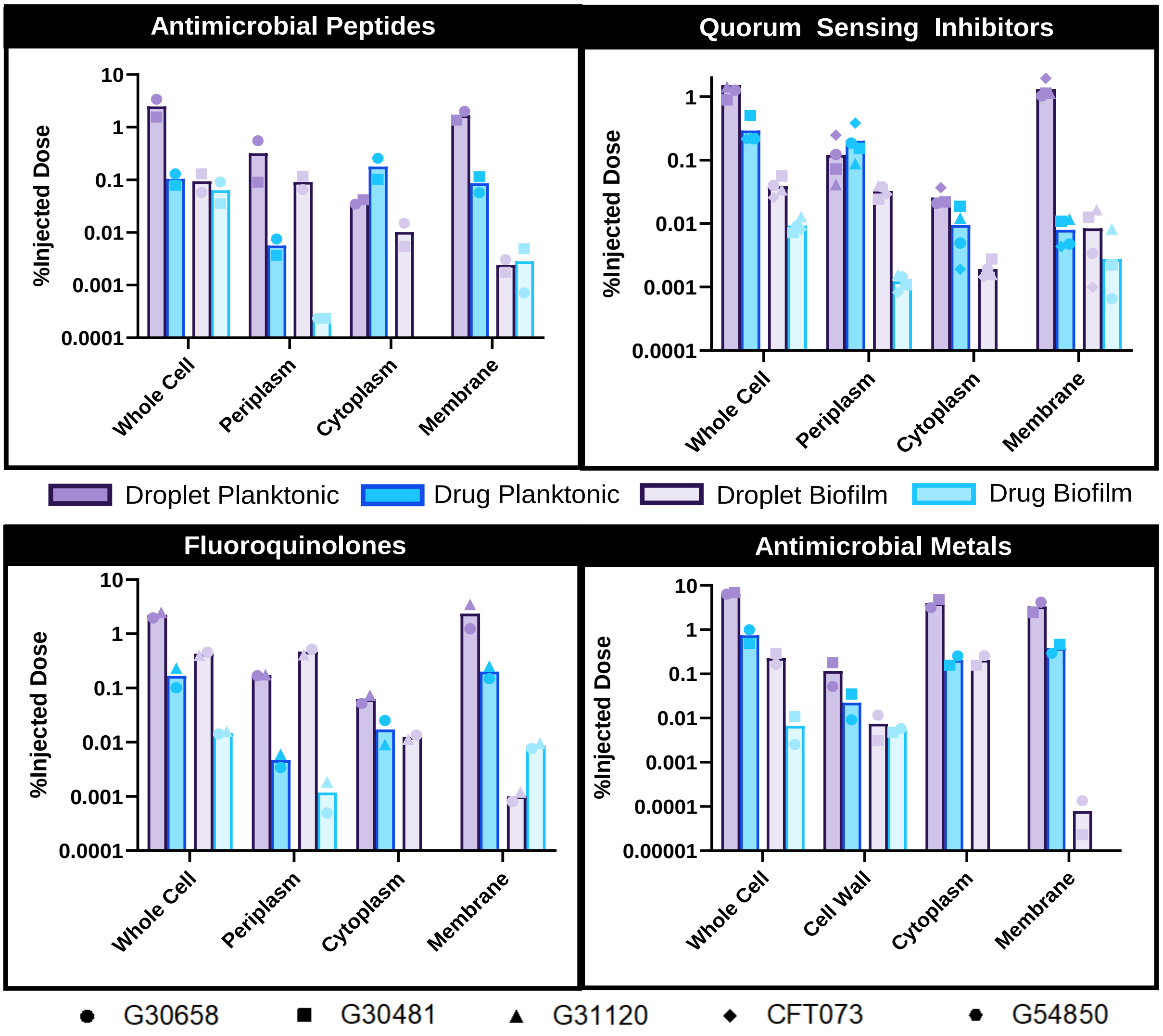

Figure S16: Raw cellular and subcellular uptake of metalated antimicrobials free and loaded into nanodroplets following administration into *E. coli* planktonic and biofilm clinical isolates as measured using ICP-MS. Symbols represent different clinical isolates, performed in triplicate.

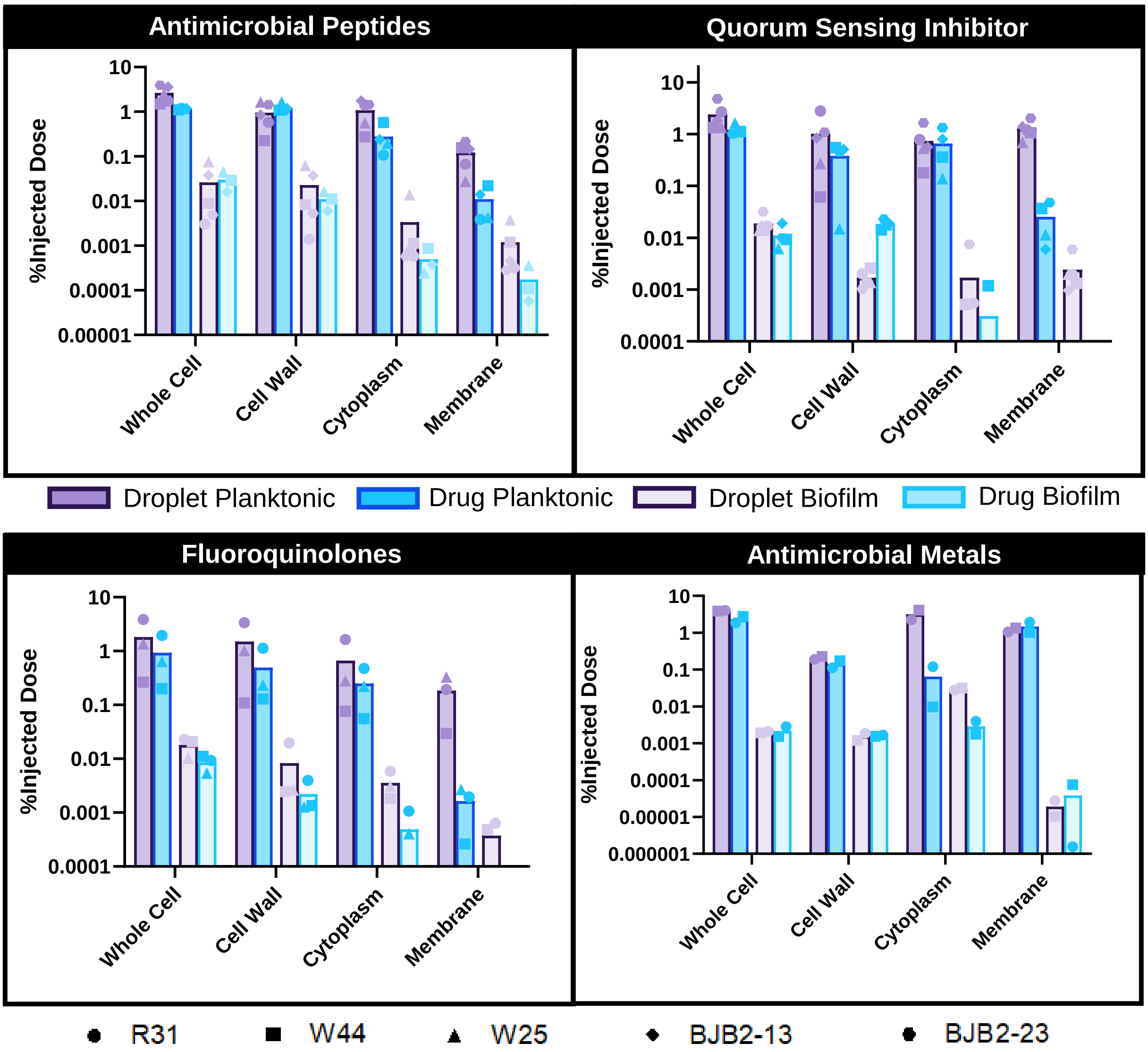

Figure S17: Raw cellular and subcellular uptake of metalated antimicrobials free and loaded into nanodroplets following administration into *S. aureus* planktonic and biofilm clinical isolates as measured using ICP-MS. Symbols represent different clinical isolates, performed in triplicate.

Figure S18: Subcellular compartment calculations for both *E. coli* and *S. aureus* planktonic and biofilm-embedded bacteria. Dimensions of subcompartments were retrieved from Matias et al.[1,2]

Number of cells at 25mL at OD 1.01 = 3.6652 x 10^8 CFU/mL * 25mL = 9.163 x 10^9 cells

E.coli
Diameter = 1.3um
Total length = 3.9um
Length cylinder = 2.6um
Thickness P = 20.99 nm +/- 2.69 = periplasmic width
Thickness M = Plasma membrane 5.84nm +/- 0.38 + Outer membrane 6.87nm +/- 1.01

Volume of subcompartments (assuming capsule – cylinder + two hemisphere)
Total volume/cell = $\pi r^2(h+4r/3)$ = 3.45 + 1.15035 = 4.60035 um^3
Total volume = 4.60 um^3 x 9.163 x 10^9 **= 42149800000um^3 = 42.15 mm^3**
Volume without Outer Membrane (r = 0.64313) = 3.34567 + 1.09807 = 4.44374 um^3
Volume Outer Membrane = 4.60035 um^3 - 4.44374 um^3 = 0.15661 um^3
Total volume Outer Membrane = 0.15661 um^3 x 9.163 x 10^9 **= 1.435 mm^3**
Volume without OM/Periplasm (r = 0.62214) = 3.16155 + 1.00868 = 4.17023 um^3
Volume Periplasm = 4.44374 um^3 - 4.17023 um^3 = 0.27351 um^3
Total volume Periplasm = 0.27351 um^3 x 9.163 x 10^9 **= 2.506 mm^3**
Volume without OM/P/PM (r = 0.6163um) = 3.10247 + 0.98054 = 4.08301 um^3
Total Volume Cytoplasm = 4.08301 um^3 x 9.163 x 10^9 = **37.413 mm^3**
Volume Plasma Membrane = 4.17023 um^3 - 4.08301 um^3 = 0.08722 um^3
Total Volume Plasma Membrane = 0.08722 um^3 x 9.163 x 10^9 **= 0.799 mm^3**

Number of cells at 25mL at OD = 1.01 = 5.94 x 10^8 CFU/mL * 25mL = 1.485 x 10^{10} cells

S Aureus
Diameter = 1.05um +/- 0.07
Thickness M = Plasma membrane 5.4nm +/- 0.4
Thickness P (cell wall) = Inner wall zone 15.8nm +/- 2.5 + Outer wall zone 19.0nm +/- 4.3
Proteoplast = 0.97um +/- 0.07

Volume of subcompartments (assuming spherical)
Total volume/cell = $4/3\pi r^3$ = 0.60613 um^3
Total volume = 0.60613 um^3 x 1.485 x 10^{10} **= 9.0010305 mm^3**
Volume without Cell Wall (r = 0.4902um) = 0.49341 um^3
Volume Cell Wall = 0.60613 um^3 - 0.49341 um^3 = 0.11272 um^3
Total Volume Cell Wall = 0.11272 um^3 x 1.485 x 10^{10} = 1.673892 mm^3
Volume without Cell Wall/Plasma Membrane (r = 0.4848um) = 0.47728 um^3
Total Volume Proteoplast = 0.47728 um^3 x 1.485 x 10^{10} = 7.087608 mm^3
Volume Plasma Membrane = 0.49341 um^3 - 0.47728 um^3 = 0.01613 um^3
Total Volume Plasma Membrane = 0.01613 um^3 x 1.485 x 10^{10} = 0.23953 mm^3

Biofilm - Number of cells removed at 33mL = 2.3 x 10^6 CFU/mL * 33mL = 7.59 x 10^7 cells

E.coli
Diameter = 1.3um
Total length = 3.9um
Length cylinder = 2.6um
Thickness P = 20.99 nm +/- 2.69 = periplasmic width
Thickness M = Plasma membrane 5.84nm +/- 0.38 + Outer membrane 6.87nm +/- 1.01

Volume of subcompartments (assuming capsule – cylinder + two hemisphere)
Total volume/cell = $\pi r^2(h+4r/3)$ = 3.45 + 1.15035 = 4.60035 um^3
Total volume = 4.60 um^3 x 7.59 x 10^7 = 34914000 um^3 = 0.349 mm^3
Volume without Outer Membrane (r = 0.64313) = 3.34567 + 1.09807 = 4.44374 um^3
Volume Outer Membrane = 4.60035 um^3 - 4.44374 um^3 = 0.15661 um^3
Total volume Outer Membrane = 0.15661 um^3 x 7.59 x 10^7 = 0.012 mm^3
Volume without OM/Periplasm (r = 0.62214) = 3.16155 + 1.00868 = 4.17023 um^3
Volume Periplasm = 4.44374 um^3 - 4.17023 um^3 = 0.27351 um^3
Total volume Periplasm = 0.27351 um^3 x 7.59 x 10^7 = 0.0208 mm^3
Volume without OM/P/PM (r = 0.6163um) = 3.10247 + 0.98054 = 4.08301 um^3
Total Volume Cytoplasm = 4.08301 um^3 x 7.59 x 10^7 = 0.31 mm^3
Volume Plasma Membrane = 4.17023 um^3 - 4.08301 um^3 = 0.08722 um^3
Total Volume Plasma Membrane = 0.08722 um^3 x 7.59 x 10^7 = 0.00662 mm^3

Number of cells removed at 33mL = 1.2 x 10^6 CFU/mL * 33mL = 3.96 x 10^7 cells

S Aureus
Diameter = 1.05um +/- 0.07
Thickness M = Plasma membrane 5.4nm +/- 0.4
Thickness P (cell wall) = Inner wall zone 15.8nm +/- 2.5 + Outer wall zone 19.0nm +/- 4.3
Proteoplast = 0.97um +/- 0.07

Volume of subcompartments (assuming spherical)
Total volume/cell = $4/3\pi r^3$ = 0.60613 um^3
Total volume = 0.60613 um^3 x 3.96 x 10^7 = 0.024 mm^3
Volume without Cell Wall (r = 0.4902um) = 0.49341 um^3
Volume Cell Wall = 0.60613 um^3 - 0.49341 um^3 = 0.11272 um^3
Total Volume Cell Wall = 0.11272 um^3 x 3.96 x 10^7 = 0.00446 mm^3
Volume without Cell Wall/Plasma Membrane (r = 0.4848um) = 0.47728 um^3
Total Volume Proteoplast = 0.47728 um^3 x 3.96 x 10^7 = 0.0189 mm^3
Volume Plasma Membrane = 0.49341 um^3 - 0.47728 um^3 = 0.01613 um^3
Total Volume Plasma Membrane = 0.01613 um^3 x 3.96 x 10^7 = 6.3875e^{-4} mm^3

1 Matias, V. R., Al-Amoudi, A., Dubochet, J. & Beveridge, T. J. Cryo-transmission electron microscopy of frozen-hydrated sections of Escherichia coli and Pseudomonas aeruginosa. *J Bacteriol* **185**, 6112-6118, doi:10.1128/jb.185.20.6112-6118.2003 (2003).
2 Matias, V. R. & Beveridge, T. J. Native cell wall organization shown by cryo-electron microscopy confirms the existence of a periplasmic space in Staphylococcus aureus. *J Bacteriol* **188**, 1011-1021, doi:10.1128/jb.188.3.1011-1021.2006 (2006).

Table of Contents

.... 1
Specify trans structure array 1
Define system parameters 2
Definition of the transmitted angles 2
Specify TPC 2
Specify P structure array (Previous SFormat structure) 3
Max Depth ToF Calculation 3
PRF Calculation 3
Set up PData structure in cartesian domain 3
Resources 4
Definition of inter buffer 4
Definition of Image Buffer 4
Windows Display 4
TW structure array 5
Specify TX structure array. 5
Vaporization 5
Delivery 5
First pulse - Negative 5
Specify TGC Waveform structure - %%need to double check double TGC structure and presets 6
Specify Receive structure 6
Receive Data Structure 6
Changed to add PIAM - see run 6
Reconstruction 7
Specify Process structure array. 7
External RF Processing 8
Specify SeqControl structure arrays. 8
Vaporization 9
Delivery 10
Acquisition SAVE DATA 12


```
cd('C:\Users\jruan\Documents\Victor\Vantage-4.8.4-2211151000');
activate;

clear all;

savedir='C:\Users\jruan\Documents\Victor\Vantage-4.8.4-2211151000\Data';
    if ~exist(savedir,'dir')
        mkdir(savedir);
    end

experimenttype = 'uti1';
filename = [datestr(now,'mm-dd-yyyy'),experimenttype];
 cd
```

Specify trans structure array

```
Trans.name = 'C5-2';
Trans.units = 'wavelengths'; % required in Gen3 to prevent default to mm units
```

```
Trans = computeTrans(Trans);  % C5-2v transducer is 'known' transducer so we
 can use computeTrans.
freq_bw = 1/3;
Trans.Bandwidth = [Trans.frequency*(1-freq_bw), Trans.frequency*(1+freq_bw)];
Trans.maxHighVolvtage = 30;
scaleFactor = 1.5;    % weight of the second acquisition wrt first one: allows
 adjusting ratio of fundamental to second harmonic in image
                    % 0=first acq only  1=equal weighting  2=second acq only
 (assuming the two cancel completely when =1)
```

Define system parameters

```
Resource.Parameters.Connector = 1;                            % Connector to
 use
Resource.Parameters.numTransmit = 128;                        % number of
 transmit channels.
Resource.Parameters.numRcvChannels = 128;                     % number of
 receive channels.
Resource.Parameters.speedOfSound = 1540;                      % set speed of
 sound in m/sec before calling computeTrans
Resource.Parameters.speedCorrectionFactor = 1.0;              % Speed
 coefficient correction
Resource.Parameters.simulateMode = 0;        % Select the simulation mode
Resource.Parameters.verbose = 3;                              % warning
 level 0:3(error,warning,status, debug)
Resource.Parameters.initializeOnly = 0;                       % set to
 initialise parameter w/0 running the hardware
Resource.Parameters.waitForProcessing = 0;
Resource.Parameters.scaleToWvl = Trans.frequency/...
    (Resource.Parameters.speedOfSound/1000);                  % MM to
 Wavelength
Resource.Parameters.ProbeThermistor = ...
    repmat(struct('enable', 0, ...
                  'threshold', 0, ...
                  'reportOverThreshold', 0), 1, 2);
%Resource.HIFU.extPwrComPortID = COM3;        %Enable HIFU power supply
%Resource.HIFU.externalHifuPwr = 1;           %Enable HIFU
%Resource.HIFU.voltageTrackP5 = 1;            %Allows use of normal short-
duration bursts from Profile 5 power supply
```

Definition of the transmitted angles

```
Custom.Delivery.NumAngles = 10;
Custom.Delivery.AngleRange = 15;
```

Specify TPC

```
TPC(1).name = 'Image';
TPC(1).maxHighVoltage = 10;
TPC(1).hv = 1.2;
TPC(1).xmitduration = 25;
TPC(2).name = 'Vaporize';
```

```
TPC(2).hv = 33.1;
```

Specify P structure array (Previous SFormat structure)

```
SampleDepthMM = 130;

SampleDepth = ceil(SampleDepthMM*Resource.Parameters.scaleToWvl);
P.aperture = (Trans.numelements)*Trans.spacing;
P.startDepth = 30;
P.endDepth = SampleDepth;
```

Max Depth ToF Calculation

```
Custom.SamplesPerWave = 1;

TOF = ceil(SampleDepthMM/1000*2/1540*1e6);
MaxAcqLength = sqrt(P.endDepth^2 + (P.aperture)^2)-P.startDepth; % Maximum
 distance Based on Verasonics calculation
wlsPer128 = Resource.Parameters.numRcvChannels/(Custom.SamplesPerWave*2);
                     % wavelengths in 128 samples for PI
numRcvSamples = 2*(wlsPer128*ceil(MaxAcqLength/
wlsPer128))*Custom.SamplesPerWave;
MaxAcqLength=numRcvSamples/(Custom.SamplesPerWave*2);  % final acquisition
 depth in wavelength
```

PRF Calculation

```
Custom.Delivery.DutyCycle = 0.1; %percentage
Custom.Delivery.PulseOn = 0.05; %ms
Custom.Vaporization.DutyCycle = 30; %percentage
Custom.Vaporization.PulseOn = 0.0032; %ms

PRF.Delivery = Custom.Delivery.PulseOn*1000/
Custom.Delivery.DutyCycle/100*10000; %Time between frames = 1ms
PRF.Vaporize = Custom.Vaporization.PulseOn*1000/
Custom.Vaporization.DutyCycle/100*10000; %Time between frames = 10.667us

Delivery.FrameRate = 1/(3*TOF*Custom.Delivery.NumAngles)*1e6; %3 pulses per
 frame for PIAM
```

Set up PData structure in cartesian domain

```
PData.PDelta = [Trans.spacing, 0, 1]; %x,y,z
PData.Size(1) = ceil(P(1).endDepth)/PData.PDelta(3);
PData.Size(2) = ceil(P(1).aperture/PData.PDelta(1));
PData.Size(3) = 1;
PData.Origin = [-
Trans.spacing*(Resource.Parameters.numRcvChannels-1)/2,0,0]; % x,y,z of upper
 lft crnr.
```

```
% No PData.Region specified, so a default Region for the entire PData array
 will be created by computeRegions.
```

Resources

```
Custom.Delivery.numAcq = 1;
Custom.Delivery.numFrames = 5;
    if rem(Custom.Delivery.numFrames,2) %must be even
        Custom.Delivery.numFrames = Custom.Delivery.numFrames + 1;
    end
Custom.Delivery.numFramesDisplay = 5; %number of frames to be displayed/will
 overwrite

if Custom.Delivery.numFramesDisplay>Custom.Delivery.numFrames,
 Custom.Delivery.numFramesDisplay = Custom.Delivery.numFrames; end
Num.skipframes = round(Delivery.FrameRate/(30*Custom.Delivery.numAcq));
    if Num.skipframes>Custom.Delivery.numFramesDisplay
        Num.skipframes = Custom.Delivery.numFramesDisplay;
    end
Resource.RcvBuffer(1).datatype = 'int16';
Resource.RcvBuffer(1).rowsPerFrame =
 numRcvSamples*Custom.Delivery.numAcq*Custom.Delivery.NumAngles;
Resource.RcvBuffer(1).colsPerFrame = Resource.Parameters.numRcvChannels;
Resource.RcvBuffer(1).numFrames = Custom.Delivery.numFrames;
```

Definition of inter buffer

```
Resource.InterBuffer(1).datatype = 'complex double'; %or complex
Resource.InterBuffer(1).numFrames = 1;
Resource.InterBuffer(1).rowsPerFrame = 1024;
Resource.InterBuffer(1).colsPerFrame = PData(1).Size(2);
```

Definition of Image Buffer

```
Resource.ImageBuffer(1).datatype = 'double';
Resource.ImageBuffer(1).numFrames = 1;
Resource.ImageBuffer(1).rowsPerFrame = PData(1).Size(1);
Resource.ImageBuffer(1).colsPerFrame = PData(1).Size(2);
```

Windows Display

```
Resource.DisplayWindow(1).Title = 'Delivery';
Resource.DisplayWindow(1).pdelta = 0.35;
ScrnSize = get(0,'ScreenSize');
DwWidth = ceil(PData(1).Size(2)*PData(1).PDelta(1)/
Resource.DisplayWindow(1).pdelta);
DwHeight = ceil(PData(1).Size(1)*PData(1).PDelta(3)/
Resource.DisplayWindow(1).pdelta);
Resource.DisplayWindow(1).Position = [200,(ScrnSize(4)-(DwHeight+150))/2, ...
 % lower left corner position
                                              DwWidth, DwHeight];
```

```
Resource.DisplayWindow(1).ReferencePt =
 [PData(1).Origin(1),0,PData(1).Origin(3)];   % 2D imaging is in the X,Z plane
Resource.DisplayWindow(1).Type = 'Verasonics';
Resource.DisplayWindow(1).numFrames = 20; %Cineloop frame, depends on gpu
 capability
Resource.DisplayWindow(1).AxesUnits = 'mm';
Resource.DisplayWindow(1).Colormap = pink(256); %or gray(256)
```

TW structure array

```
TW(1).type = 'parametric';
TW(1).Parameters = [Trans.frequency,.99,158,-1];   % A, B, C, D

% FOCUS TRANSMISSION
TW(2).type = 'parametric';
TW(2).Parameters = [Trans.frequency,.99,18,-1];   % A, B, C, D
```

Specify TX structure array.

```
Custom.Focus.steps = 30;

TX = repmat(struct('waveform', 1, ...
                   'Apod', ones(1,Trans.numelements), ...
                   'Delay', zeros(1,Trans.numelements),...
                   'peakCutOff', 0.1,...
                   'peakBLMax', 40.50), 1,Custom.Delivery.NumAngles
+Custom.Focus.steps);
```

Vaporization

```
for n = 1:Custom.Focus.steps
        TX(n).waveform = 2;
        TX(n).FocalPt = [(-28.8+(50/(Custom.Focus.steps-1))*(n-1)), 0.2,
 200.0];
end
clearvars k n;
```

Delivery

```
k = Custom.Focus.steps;
for n = 1:Custom.Delivery.NumAngles
```

First pulse - Negative

```
    TX(n+k).waveform = 1;
    TX(n+k).FocalPt = [(-28.8+(50/(Custom.Delivery.NumAngles-1))*(n-1)), 0.2,
 200.0];
    %TX(n+k).Delay = computeTXDelays(TX(n+k),'TOAE');
end
clearvars k k1 n;
```

Specify TGC Waveform structure - %%need to double check double TGC structure and pre-sets

```
Preset.TGC(1,:)=[613,660,760,850,945,1023,1023,1023];   % does affect RF data
Preset.TGC(2,:)=[613,660,760,850,945,1023,1023,1023];   % does affect RF data

TGC = repmat(struct('CntrlPts',[],...
    'rangeMax',[],....
    'Waveform',[]),1,size(Preset.TGC,1));

for i = 1:size(Preset.TGC,1)
    TGC(i).CntrlPts = Preset.TGC(i,:);
    TGC(i).rangeMax = P(1).endDepth;
    TGC(i).Waveform = computeTGCWaveform(TGC(i));
end
clearvars i;
```

Specify Receive structure

```
BPF = [-0.00055 +0.00000 +0.00137 +0.00000 -0.00092 +0.00000 -0.00290 ...
       +0.00000 +0.01266 +0.00000 -0.03021 +0.00000 +0.05527 +0.00000 ...
       -0.08475 +0.00000 +0.11307 +0.00000 -0.13361 +0.00000 +0.14111];
LPC = [-0.00058 -0.00577 -0.01303 -0.01913 -0.01923 -0.00842...
       +0.01605 +0.05313 +0.09723 +0.13943 +0.16989 +0.18097];

% Specify Receive structure arrays.
Receive = repmat(struct('Apod',
 ones(1,Resource.Parameters.numRcvChannels), ...
                        'startDepth', P.startDepth, ...
                        'endDepth', P.startDepth +
 wlsPer128*ceil(MaxAcqLength/wlsPer128), ...
                        'TGC', 1, ...
                        'bufnum', 1, ...
                        'framenum', 1, ...
                        'acqNum', 1, ...
                        'InputFilter',BPF, ...
                        'LowPassCoef',LPC, ...
                        'samplesPerWave', Custom.SamplesPerWave, ...
                        'mode', 0, ...
                        'callMediaFunc',
 0),1,Custom.Delivery.NumAngles*Custom.Delivery.numAcq*Custom.Delivery.numFrames);
```

Receive Data Structure

Changed to add PIAM - see run

```
for i = 1:Custom.Delivery.numFrames
    k = Custom.Delivery.NumAngles*(i-1)*Custom.Delivery.numAcq;
```

```
    for j = 1:Custom.Delivery.NumAngles*Custom.Delivery.numAcq
        Receive(k+j).bufnum = 1;
        Receive(k+j).Apod= Receive(k+j).Apod;
        Receive(k+j).TGC = 1;
        Receive(k+j).callMediaFunc = 1;
        Receive(k+j).framenum = i;
        Receive(k+j).acqNum = j;
        Receive(k+j).mode = 0;
    end
end
clearvars k2 k k1 k3 j i;
```

Reconstruction

Specify Recon structure arrays.

```
s = Custom.Delivery.NumAngles*Custom.Delivery.numAcq;
Recon = struct('senscutoff', 0.5, ...
               'pdatanum', 1, ...
               'IntBufDest', [1,1], ...
               'ImgBufDest', [1,-1], ...
               'rcvBufFrame',-1, ...
               'RINums',1:s);

% Define ReconInfo structures.
ReconInfo = repmat(struct('mode', 'accumIQ', ...  % default is to accumulate
 IQ data.
                   'txnum', 1, ...
                   'rcvnum', 1, ...
                   'regionnum', 1), 1, s);
% - Set specific ReconInfo attributes.
ReconInfo(1).mode = 'replaceIQ';
for j = 1:s % For each row in the column
    ReconInfo(j).txnum = j;
    ReconInfo(j).rcvnum = j;
end
ReconInfo(s).mode = 'accumIQ_replaceIntensity';  % accumulate and detect
clearvars s j;
```

Specify Process structure array.

```
pers = 0;
Process(1).classname = 'Image';
Process(1).method = 'imageDisplay';
Process(1).Parameters = {'imgbufnum',1,...   % number of buffer to process.
                         'framenum',-1,...   % frame number in src buffer (-1
 => lastFrame)
                         'pdatanum',1,...    % number of PData structure to
 use (defines output figure)
                         'pgain',20,...            % pgain is image processing
 gain
                         'reject',2,...      % reject level
                         'persistMethod','simple',...
```

```
                          'persistLevel',pers,...
                          'interpMethod','4pt',...  %method of interp. (1=4pt)
                          'grainRemoval','medium',...
                          'processMethod','reduceSpeckle1',...
                          'averageMethod','runAverage3',...
                          'compressMethod','power',...
                          'compressFactor',40,...
                          'mappingMethod','full',...
                          'display',1,...      % display image after processing
                          'displayWindow',1};
```

External RF Processing

```
Process(2).classname = 'External';
Process(2).method = 'RFplotandsave_C52'; % Mike RF processing
Process(2).Parameters ={'srcbuffer','receive',... %process receive buffer
                         'srcbufnum',1,...
                         'srcframenum',-1,... %process last frame
                         'dstbuffer','none'};
```

Specify SeqControl structure arrays.

- TOF for all acquisition

```
SeqControl(1).command = 'timeToNextAcq';        % time between AM / Focus
 pulse
SeqControl(1).argument = 228;
SeqControl(2).command = 'timeToNextAcq';        % time between AM frames
SeqControl(2).argument = 228;
SeqControl(3).command = 'timeToNextAcq';
SeqControl(3).argument = (PRF.Delivery);

SeqControl(4).command = 'returnToMatlab';       % Return to Matlab

SeqControl(5).command = 'jump';                 % jump back to TPC initial
SeqControl(5).argument = 1;
SeqControl(6).command = 'setTPCProfile'; % -- Change to Profile 1 (Imaging)
SeqControl(6).condition = 'immediate';
SeqControl(6).argument = 1;
SeqControl(7).command = 'setTPCProfile'; % -- Change to Profile 2
 (Vaporization)
SeqControl(7).condition = 'immediate';
SeqControl(7).argument = 2;
% set a delay between imaging and destruction (changing TPC)
% (value*200nsec; max. value is 2^25 - 1. (2^25 - 1)*200ns=6.7 sec)
% value = X(ns)/200
noopTime=1e-3; %1ms (max=104.8ms)
SeqControl(8).command = 'noop';
SeqControl(8).argument = noopTime/200e-9;
SeqControl(8).condition = 'Hw&Sw';

% Syn hardware and software
SeqControl(9).command = 'sync';
```

```
SeqControl(9).argument = 800000;

% - Set loop count.
SeqControl(10).command = 'loopCnt';
SeqControl(10).argument = 3;
SeqControl(10).condition = 'counter1';
% - Jump to test loop count.
SeqControl(11).command = 'jump';                     % jump to nTest.
SeqControl(11).argument = [];
% - Jump back to start of Destruction
SeqControl(12).command = 'loopTst';                  % jump back to start of
 destruction.
SeqControl(12).argument = [];
SeqControl(12).condition = 'counter1';
SeqControl(13).command = 'timeToNextAcq';
SeqControl(13).argument = (PRF.Vaporize);
SeqControl(14).command = 'stop';
SeqControl(15).command = 'triggerOut';
SeqControl(15).condition = 'syncNone';
SeqControl(16).command = 'loopCnt';
SeqControl(16).argument = 3;
SeqControl(16).condition = 'counter2';
% - Jump to test loop count.
SeqControl(17).command = 'jump';
SeqControl(17).argument = [];
SeqControl(18).command = 'loopTst';
SeqControl(18).argument = [];
SeqControl(18).condition = 'counter2';

nsc = length(SeqControl)+1; % nsc is count of SeqControl objects
```

Vaporization

```
n = 1;
% Change TPC for destruction
Event(n).info = 'Change Vaporization TPC';
Event(n).tx = 0;        % no TX
Event(n).rcv = 0;       % no Rcv
Event(n).recon = 0;     % no Recon
Event(n).process = 0;
Event(n).seqControl = [7,8];
n = n+1;

Event(n).info = 'Vaporization Loop Count';
Event(n).tx = 0;
Event(n).rcv = 0;
Event(n).recon = 0;
Event(n).process = 0;
Event(n).seqControl = 10;
n = n+1;

Event(n).info = 'Jump to loop count test.';
Event(n).tx = 0;
```

```
Event(n).rcv = 0;
Event(n).recon = 0;
Event(n).process = 0;
Event(n).seqControl = 11;
n = n+1;

SeqControl(12).argument = n; % Sets the jump event no. for start of accums.
            Event(n).info = 'Vaporization';
            Event(n).tx = 1;   % use next TX structure.
            Event(n).rcv = 0;
            Event(n).recon = 0;      % no reconstruction.
            Event(n).process = 0;    % no processing
            Event(n).seqControl = [13,15];
            n = n+1;

        for i = 2:Custom.Focus.steps

            Event(n).info = 'Vaporization';
            Event(n).tx = i;   % use next TX structure.
            Event(n).rcv = 0;
            Event(n).recon = 0;      % no reconstruction.
            Event(n).process = 0;    % no processing
            Event(n).seqControl = 13;
            n = n+1;
        end
        Event(n-1).seqControl = 13; % Replace last event's seqControl for
 frame time
SeqControl(11).argument = n; % Set jmp event for the 'jmp-to-test' SeqControl.

Event(n).info = 'Vaporization Loop Test';
Event(n).tx = 0;
Event(n).rcv = 0;
Event(n).recon = 0;
Event(n).process = 0;
Event(n).seqControl = 12;
n = n+1;

% Change TPC for Imaging
Event(n).info = 'Change Imaging TPC';
Event(n).tx = 0;        % no TX
Event(n).rcv = 0;       % no Rcv
Event(n).recon = 0;     % no Recon
Event(n).process = 0;
Event(n).seqControl = [6,8,9];
n = n+1;
```

Delivery

```
nTPCDelivery = n;
SeqControl(8).argument = nTPCDelivery;

for i = 1:Custom.Delivery.numFramesDisplay
    k = (i-1)*Custom.Delivery.numAcq*Custom.Delivery.NumAngles;
```

```
    for h=1:Custom.Delivery.numAcq
        l=Custom.Delivery.NumAngles*(h-1); % We save the data at the same rcv
of the first half aperture
        k1 = Custom.Delivery.NumAngles*Custom.Delivery.numAcq;
        ktx = Custom.Focus.steps;
        ktx1 = ktx+Custom.Delivery.NumAngles;
            Event(n).info = 'Delivery Negative';
            Event(n).tx = 1+ktx;         % use 1st TX structure.
            Event(n).rcv = l+k+1;      % use 1st Rcv structure.
            Event(n).recon = 0;      % no reconstruction.
            Event(n).process = 0;    % no processing
            Event(n).seqControl = [1,15]; % time between syn. aper. acqs.
            n = n+1;
        for j = 2:Custom.Delivery.NumAngles
            Event(n).info = 'Delivery Negative';
            Event(n).tx = j+ktx;         % use 1st TX structure.
            Event(n).rcv = l+k+j;      % use 1st Rcv structure.
            Event(n).recon = 0;      % no reconstruction.
            Event(n).process = 0;    % no processing
            Event(n).seqControl = 1; % time between syn. aper. acqs.
            n = n+1;
        end
    end

    % Replace last Event's seqControl value.
    Event(n-1).seqControl = [3,nsc]; % time between frames, SeqControl struct
defined below.
    SeqControl(nsc).command = 'transferToHost';
    nsc = nsc + 1;

    if floor(i/Num.skipframes) == i/Num.skipframes
        Event(n).info = 'Reconstruction';
        Event(n).tx = 0;         % no transmit
        Event(n).rcv = 0;        % no rcv
        Event(n).recon = 0;      % reconstruction
        Event(n).process = 0;    % processing

        Event(n).seqControl=[4,nsc,nsc+1];

        SeqControl(nsc).command='waitForTransferComplete';
        SeqControl(nsc+1).command='markTransferProcessed';
        SeqControl(nsc).argument=nsc-1;
        SeqControl(nsc+1).argument=nsc-1;

        nsc = nsc + 2;
        n = n+1;

        Event(n).info = 'RF Transfer';
        Event(n).tx = 0;         % no transmit
        Event(n).rcv = 0;        % no rcv
        Event(n).recon = 0;      % reconstruction
        Event(n).process = 2;    % processing
        Event(n).seqControl= 0;
        n = n+1;
```

```
    end
end
```

Acquisition SAVE DATA

```
Event(n).info = 'Complete Loop Count';
Event(n).tx = 0;
Event(n).rcv = 0;
Event(n).recon = 0;
Event(n).process = 0;
Event(n).seqControl = 16;
n = n+1;

Event(n).info = 'Jump to loop count test.';
Event(n).tx = 0;
Event(n).rcv = 0;
Event(n).recon = 0;
Event(n).process = 0;
Event(n).seqControl = 17;
n = n+1;

SeqControl(18).argument = 1; % Sets the jump event no. for start of accums.

SeqControl(17).argument = n; % Set jmp event for the 'jmp-to-test' SeqControl.

Event(n).info = 'Complete Loop Test';
Event(n).tx = 0;
Event(n).rcv = 0;
Event(n).recon = 0;
Event(n).process = 0;
Event(n).seqControl = 18;
n = n+1;

Event(n).info = 'Stop';
Event(n).tx = 0;
Event(n).rcv = 0;
Event(n).recon = 0;
Event(n).process = 0;
Event(n).seqControl = 14;
n = n+1;
```

Save all the structures to a .mat file.

```
save(filename);
VSX;
return
```

Published with MATLAB® R2022b
